# 3D printed custom gas cam for race bike application using Progrip® lock on grips mod.708

**DOI:** 10.12688/f1000research.125184.1

**Published:** 2022-10-11

**Authors:** Patrich Ferretti, Elena Fusari, Giulia Alessandri, Marco Freddi, Daniela Francia

**Affiliations:** 1 Department of Industrial Engineering, University of Bologna, Bologna, Italy, 40136, Italy

**Keywords:** MSLA, water washable resin, lattice structure, nTopology, Design for additive manufacturing, Stereolithography tooling, Shape optimization, CAD/CAM

## Abstract

Background: The “drive by wire” mechanism for managing the throttle is not applied to every modern motorcycle, but it is often managed through a steel wire. Here, there is a cam on the throttle control. Its shape allows the throttle opening to be faster or slower and its angle of rotation, required for full opening, to be greater or less. The maximum angle a rider’s wrist can withstand depends on numerous musculoskeletal mobility factors, often limited by falls or surgery.

Methods: Using a Progrip knob with interchangeable cams allows the customization of a special cam profile, to ensure the best engine response to throttle rotation and ergonomics for the rider. The use of FEA software and lattice structures, allows to realize a lightweight and efficient design, targeted for fabrication with additive manufacturing technologies.

Results: The cam was manufactured by exploiting MSLA technology. Finally, a dimensional inspection procedure was performed before assembly. The main result is to have obtained a lighter and cheaper component than the original.

Conclusions: This study has allowed the design of a mechanical component consisting of innovative shape, light weight, and ergonomics. Furthermore, it demonstrates the effectiveness in the use of lattice structures to enable weight optimization of a component while minimizing the increase in its compliance.

## Introduction

The Additive Manufacturing technology is now a days the only one that can guarantee the realization of custom components at reasonable cost. The component can be designed by taking advantage of the latest structural optimization software and light weighting possibilities. 3D printing offers the possibility of fabricating components in a variety of materials from the world of polymeric materials to metals. Talking about the design of components with “lattice” structures additive manufacturing automatically becomes the only one that can be considered ( Pan *et al.*, 2020). Many recently developed geometries have the distinction of being weight-optimized through the use of special internal “lattice” structures ( Gibson, 1989; Tamburrino *et al.*, 2018; Tao & Leu, 2016). These are merely internal ramifications of the geometry that can provide good stiffness while leaving numerous gaps to minimize the weight of the final structure ( Messner, 2016). The main advantage offered is the fact that the external shape of the component’s geometry may not varies in any way. That is a rather important factor considering that the shape of the component is often the main design constraint to be respected. The cells characterizing the internal patterns of such geometries can consist of simple geometries, such as hexahedra, tetrahedra, octets ( Deshpande *et al.*, 2001) or classical hexagonal honeycombs, or more complex surfaces governed by mathematical equations in 3D space ( du Plessis *et al.*, 2018). A classic example is the Gyroid structure ( Khaderi *et al.*, 2014), but many others exist, such as Neovius cells ( Khan & Abu Al-Rub, 2017), Lidinoid and Schwarz ( Shin *et al.*, 2012). These belong to “TPMS” (Triply Periodic Minimal Surface) cells category, much deployed in this design context ( Al-Ketan & Abu Al-Rub, 2019; Gandy *et al.*, 2001; Jung *et al.*, 2006; Savio *et al.*, 2019).

### MSLA (Mashed Stereolithography) Resin Printing

Stereolithography (SLA) is a printing technology patented in the 1980s that uses a laser to make components. The laser is focused on the transparent bottom of a photosensitive surface of a container filled with resin. The resin, sensitive to ultraviolet light, cures and solidifies only in the areas targeted by the laser forming a layer of material. Resins used with MSLA technology are usually thermoset polymers, these materials can exploit good mechanical properties and are used in many contexts in industry ( Croccolo *et al.*, 2019). The process is repeated until the component is created. This process offers multiple advantages, ranging from the high isotropy of the printed component combined with the high surface finish and high level of detail. Nowadays, SLA technology is seeing several evolutions, aimed to reduce the cost significantly and make it more accessible to the public, while still preserving the advantages. One of these is “MSLA” (Masked Stereolithography) whose main advantage over the older “SLA” lies in the timing for making parts. Here, in fact, it’s used an LCD screen inside the printer, consisting of pixels whose quantity directly influences the resolution of the manufactured part. The screen performs the function of a mask for ultraviolet light forming the 2D drawing of the layer to be printed thanks to the activation or deactivation of the pixels. In this way, every single point of a layer photo-polymerizes simultaneously, without the need of multiple laser-performed paths as in the case of SLA. The resin printing technologies is excellent for lattice structure realization, easily succeeding in making the beams that compose the reticulum and capturing even the smallest details.

### Customization and 3D printed parts

The need of customised components is of great interest in numerous fields, ranging from biomedical ( Frizziero, Santi, Leon-Cardenas, Donnici, Liverani, Napolitano, *et al.*, 2021; Frizziero, Santi, Leon-Cardenas, Donnici, Liverani, Papaleo, *et al.*, 2021) to mechanical engineering especially the automotive field. For several years now there have been dedicated departments within the companies themselves to meet specific customer requirements ( Fantini *et al.*, 2016; Zhang *et al.*, 2021). Before the advent of Additive Manufacturing, a “one-off” mechanical component was manufactured using one or more “traditional” technologies still widely used in industry today. This entailed very high and, in some cases, unsustainable costs because they were not justified by the production of a single component. In particular, the level of customisation that can be achieved through the use of 3D printing is unattainable by any other technologies. This makes the additive manufacturing extremely competitive, both in terms of cost and of the complexity of the geometries that can be produced. Many examples in the literature demonstrate how additive manufacturing is the winning choice. The applications go from the aerospace sector ( Moon *et al.*, 2015; Totaro & Gürdal, 2009; Vasiliev & Razin, 2006; Zhu *et al.*, 2015) to the medical-health sector cited, from the automotive sector ( Bacciaglia *et al.*, 2020a; Yin *et al.*, 2018) to the entertainment sector ( Bacciaglia *et al.*, 2020b).

## Part modelling process and lattice creation

**Figure 1.**
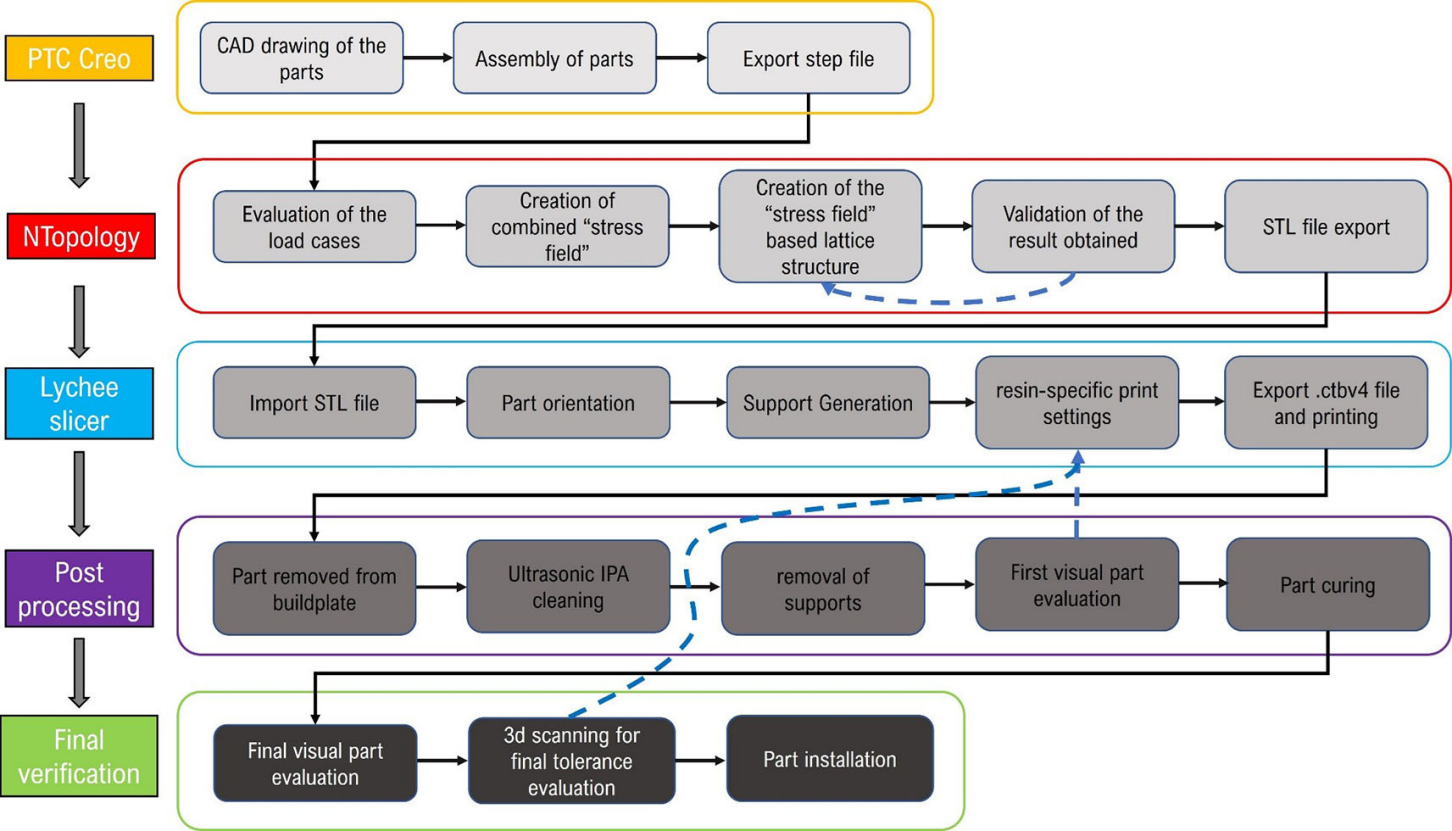
Project’s workflow.

The design of the components is conditioned by various factors: the production process adopted (resin printing); the perfect intercompatibility with respect to the Progrip mod.708 grip; the standard gas control scroll and finally the geometry required by the rider. The workflow followed is shown in Figure 1.

**Figure 2.**
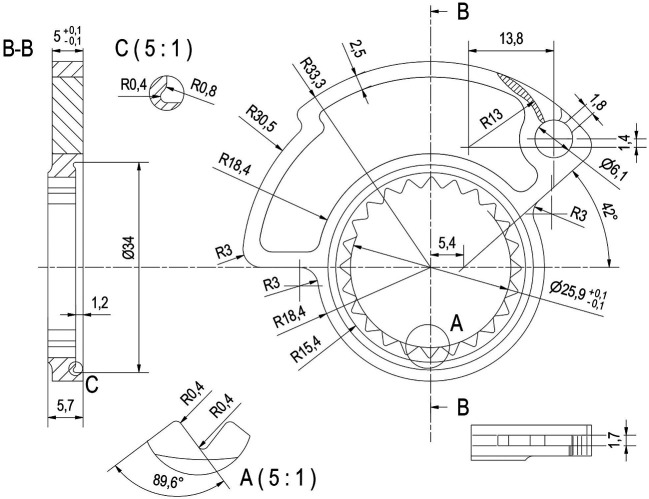
2D drawing of the cam.

The starting point is the CAD creation of an initial cam model that had similar overall dimensions to those provided in the Progrip kit, but with a custom profile, as can be seen in Figure 2. The external profile of the cam is slightly larger in diameter than the cam supplied by Progrip, allowing a complete opening of the throttle valve in less than 90° of rotation of the grip command. The cam supplied as standard with Progrip knob requires an angle greater than 90° and in our specific case, due to a reduced joint mobility of the wrist, does not allow the rider to fully open the throttle.

**Figure 3.**
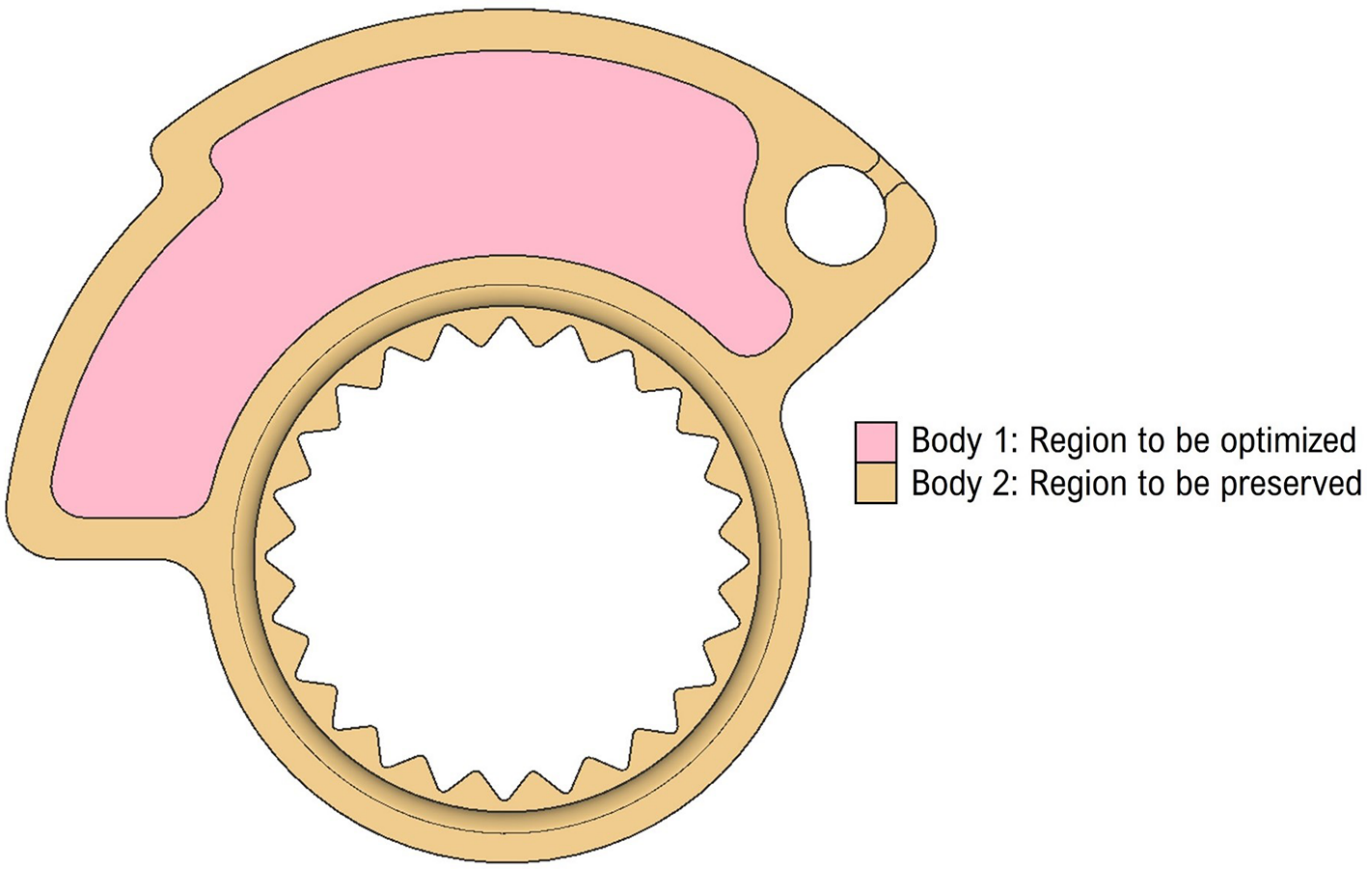
Region to be optimised and region to be preserved.

The cam was then later divided into two separate bodies as can be seen from Figure 3. A body that is the unmodifiable geometry and the other one (central part) in which apply lattice optimization to minimize the material used during printing and, at the same time, ensure the maximum stiffness values and required performance. The two bodies were exported as an assembly file in STEP format.

**Figure 4.**
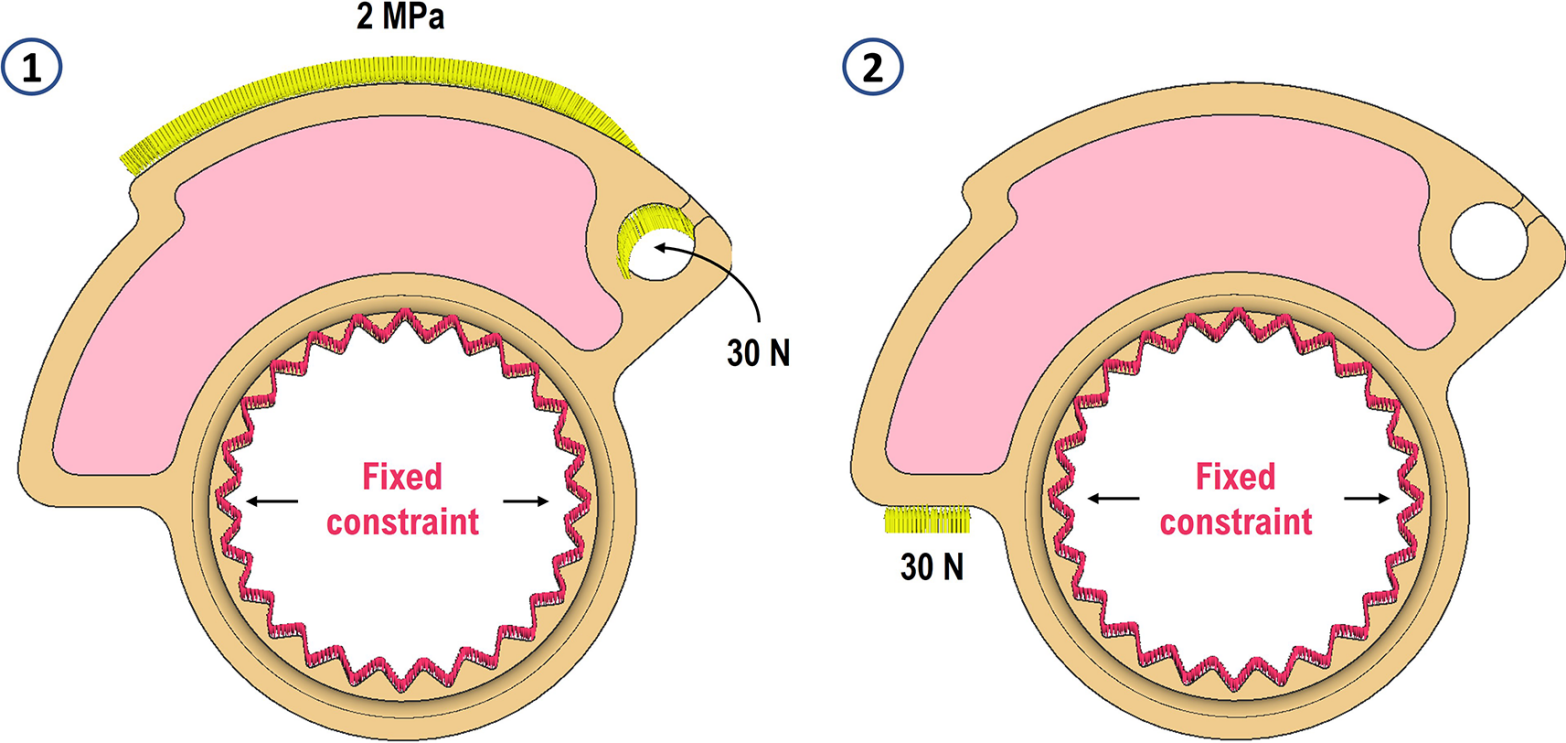
Load cases.

The geometry is then imported into nTopology, software dedicated to the design of lattice or topology-optimized components and subsequent structural verification. The component model is imported as an assembly, divided into two separate volumes. One is intended to remain unchanged at the end of the design process; the other is to be lightweighted with a lattice structure. The process leading to the final generation and export of a differentiated density lattice structure is characterized by five distinct steps as shown in Figure 1. Within the nTopology software, two simulations were setup that share the same fixed constraint on the inside of the cam but differentiated loads. The first represents the moment of maximum throttle opening, thus with the throttle control fully rotated, in which the force is generated by the return spring of the throttle and exerted on the cam through a steel wire. The second load case simulates the free release of the throttle with consequent impact of the cam end-stop on the throttle scroll due to the return spring. For the first load case 30 N were imposed inside the eyelet acting in a tangent direction to the termination of the steel wire fixed to the cam. To simulate the contact pressure between the cam and the steel wire, 2 MPa of pressure was added in an equally distributed manner along an area that resembled the dimension of the wire. For the second load case, however, 30 N of force normal to the contact face of the accelerator nut were applied to represent the effect of un uncontrolled released of the gas grip, with that part of the cam that works as an endstop. The two load cases can be seen in Figure 4. For the mesh, 0.5 mm maximum allowable length per tetrahedral element was imposed, and 0.01 mm maximum allowable gap between the actual shape of the component and the mesh. A growth rate of 2 was also imposed. These conditions result in the generation of 973431 elements and 200049 nodes. The contact between the two volumes was defined and set as a perfect bonding (Structural Bonded Contact in nTopology). Isotropic properties were assigned for the material as given below: Young’s modulus of 1930 MPa (average value compared with the properties stated in Table 1) and Poisson’s coefficient of 0.38. A material with linear-elastic behaviour was then assumed, an assumption later confirmed by the very small deformations undergone by the component as a result of the loads and also during operation. The two different structural simulations were performed sharing the same mesh and then were stored the two displacement and stress scalar fields.

**Table 1.** Mechanical properties of the resin used for printing the prototype.

Parameters	Value	Unit
Viscosity	150-300	MPa*s
Density	1.05-1.25	g/cm ^3^
Flexural Modulus	1682-2175	MPa
Flexural Strength	40-70	MPa
Heat Distortion Temperature	80	C°
Linear Shrinkage Rate %	1.05-1.35	/
Tensile Strength	30-48	MPa
Tensile Modulus	1779-2385	MPa
Elongation at Break	11-20	%
Poisson Ratio	0.38	/
Harness (Shore D)	70-80	/
Density after cured	1.09-1.18	g/cm ^3^
Notched impact Strength	41-48	J/m

**Figure 5.**
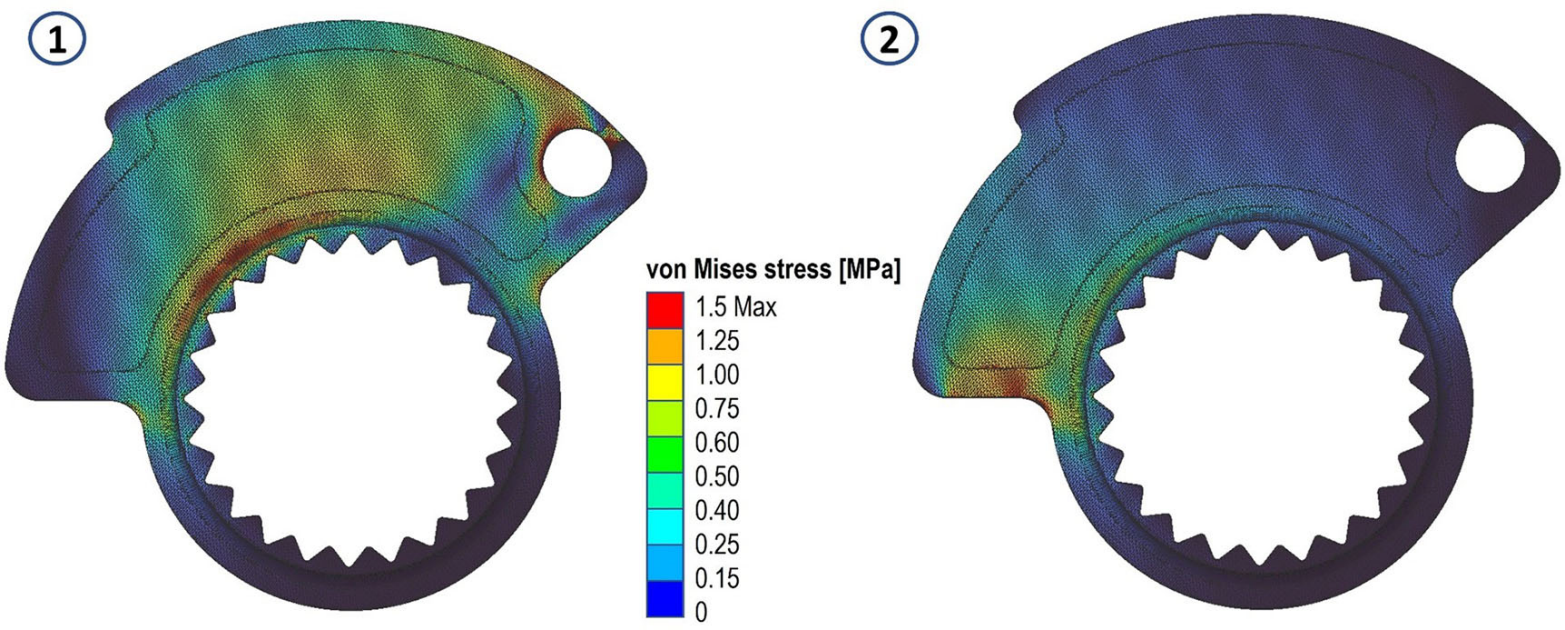
Von Mises stress evaluated for the two analyses.

**Figure 6.**
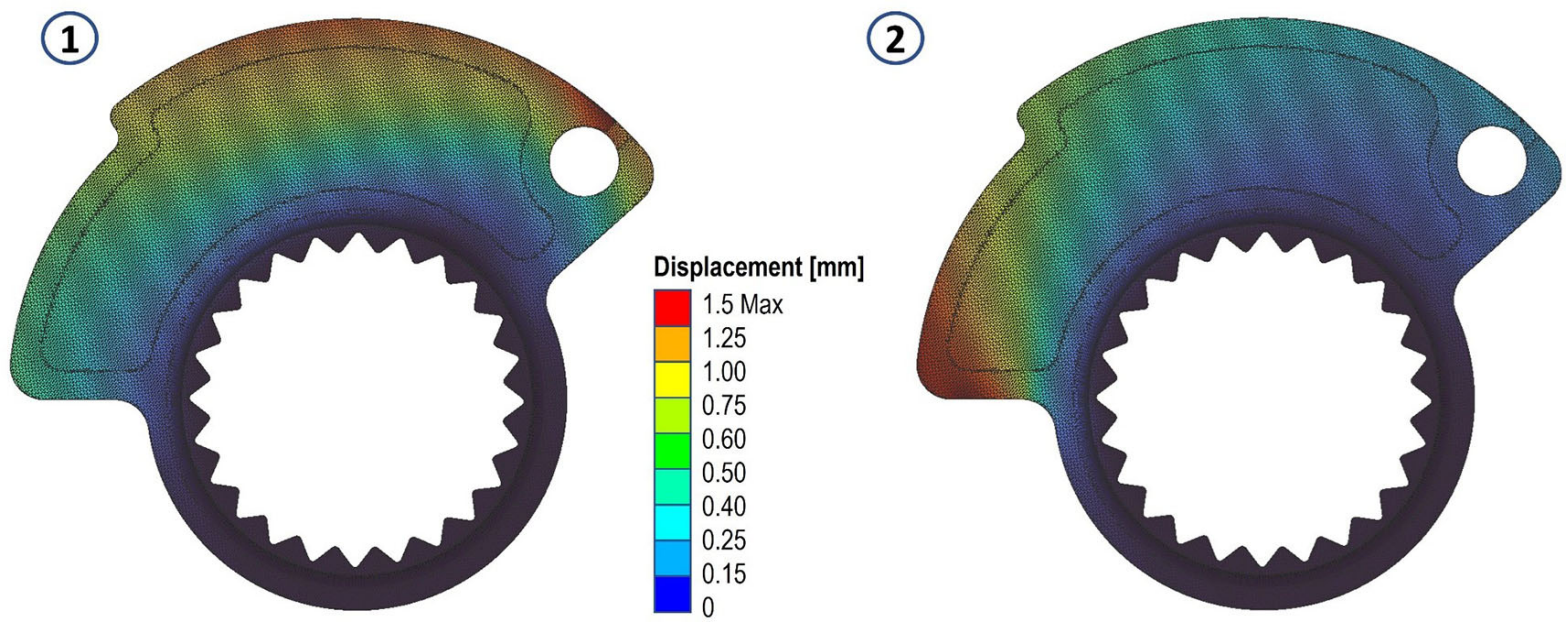
Displacements evaluated for the two analyses.

The results in terms of stress and displacements are shown in Figure 5 and Figure 6. We then proceeded to define a scalar field (Field from Point Map) as a function from R3 to R1, which takes as input the spatial position (x, y, z) of a node in the component mesh and returns as output the von Mises stress value evaluated at that node.

Having to deal with two distinct load cases and two stress fields, we opted to define a final overall stress field, in which each node in the domain is assigned the highest stress value resulting in a comparison of the values in that specific node of the two fields. This allows for any stress peaks present on either load case to be taken into account. If an average of the values of the two fields had been used, this would not have been possible.

**Figure 7.**
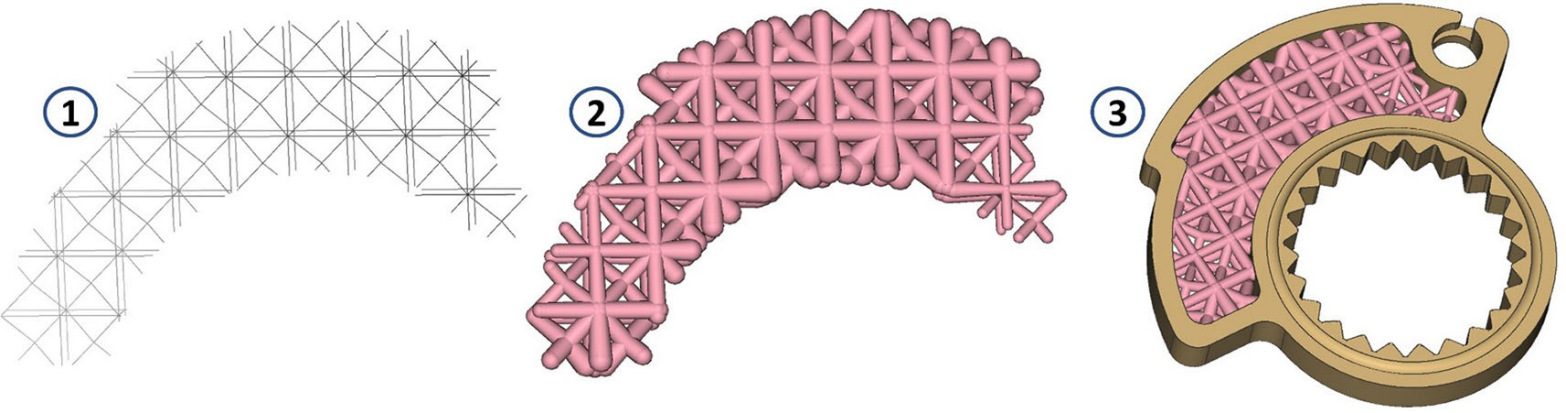
Sequence of steps constructed from the definition of the lattice grid (1) to its thickening based on the stress field (2), to the creation of the final part ready to be validated (3).

To arrive at a final design capable of respecting the density variation in the component, which is directly proportional to the stress variation, it is then necessary to define the type of cell to be used and the dimension of the trusses. The choice was to use the Tet-oct vertex centroid type, with a cell size equal to 7 mm. The lattice thus defined is trimmed so that it remains included in the volume intended for lattice optimization (1); then, we proceed by removing the “floating beams,” elements disconnected from the remaining lattice defined in that volume. At that point, we proceed by defining the thickness variability (Thicken Lattice) according to the stress field. In the case under consideration, the minimum stress value was assigned a thickness of 0.8 mm, and the maximum value of 2.5 mm. These values were defined following an optimization loop as can be seen by the blue arrow in Figure 1. The goal was to minimize the volume of material use, trying to contain stress and deformations at the same time. The value of 0.8 mm represents the minimum beam thickness achievable with this resin. This means that going below this thickness does not allow the realization of the truss beams. A useful representation for understanding these last design steps is offered in Figure 7.

Simulating again the behaviour of the component is fundamental, since the removal of material causes a decrease in its overall stiffness, and the presence of connections between trusses, within the lattice zone, can lead to localized stress peaks.

In order to verify the obtained structure, a new mesh must be defined. There are two possible ways to proceed. One can proceed with a very dense global mesh, as it will have to capture the details of each part of the variable-thickness lattice, at the expense of more computational time required for meshing and simulation, yet succeeding in capturing any localized effects. The alternative, in the case of an isotropic material, is to use a mesh in which each piece of the lattice consists of a finite “beam” type element. In this case the possibility of evaluating the effect of localized stresses is lost, but it is possible to predict deformations accurately enough and quickly. This is not so true for internal stresses, of which you get in output a set of values that are approximate. It can be a valuable tool in the case of making components with designs dominated by stiffness to have a quick evaluation of the results.

In the present case, the first choice was made. The maximum element size of the new mesh was set to 0.1 mm, and a total of 1913956 elements corresponding to 427668 nodes were generated. The simulations of the same load cases illustrated above were performed.

**Figure 8.**
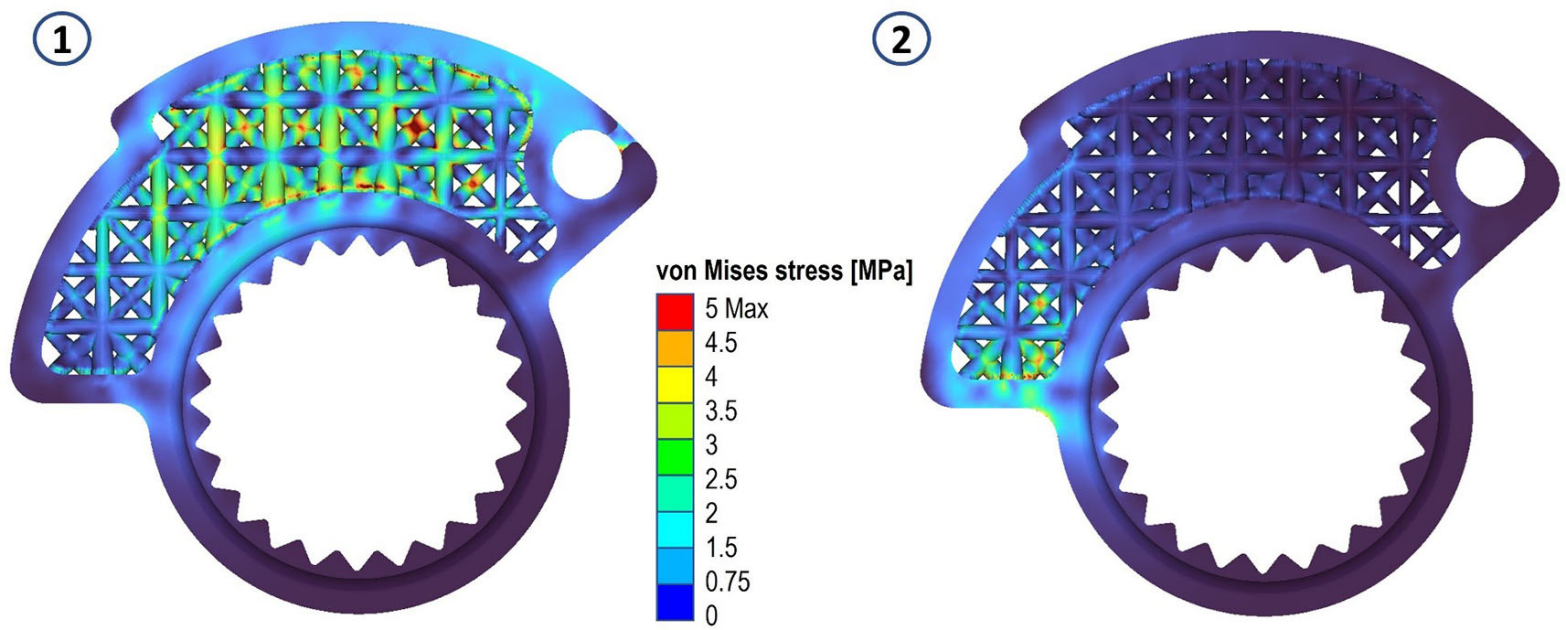
Von Mises stress for the final structures, case 1 and 2.

**Figure 9.**
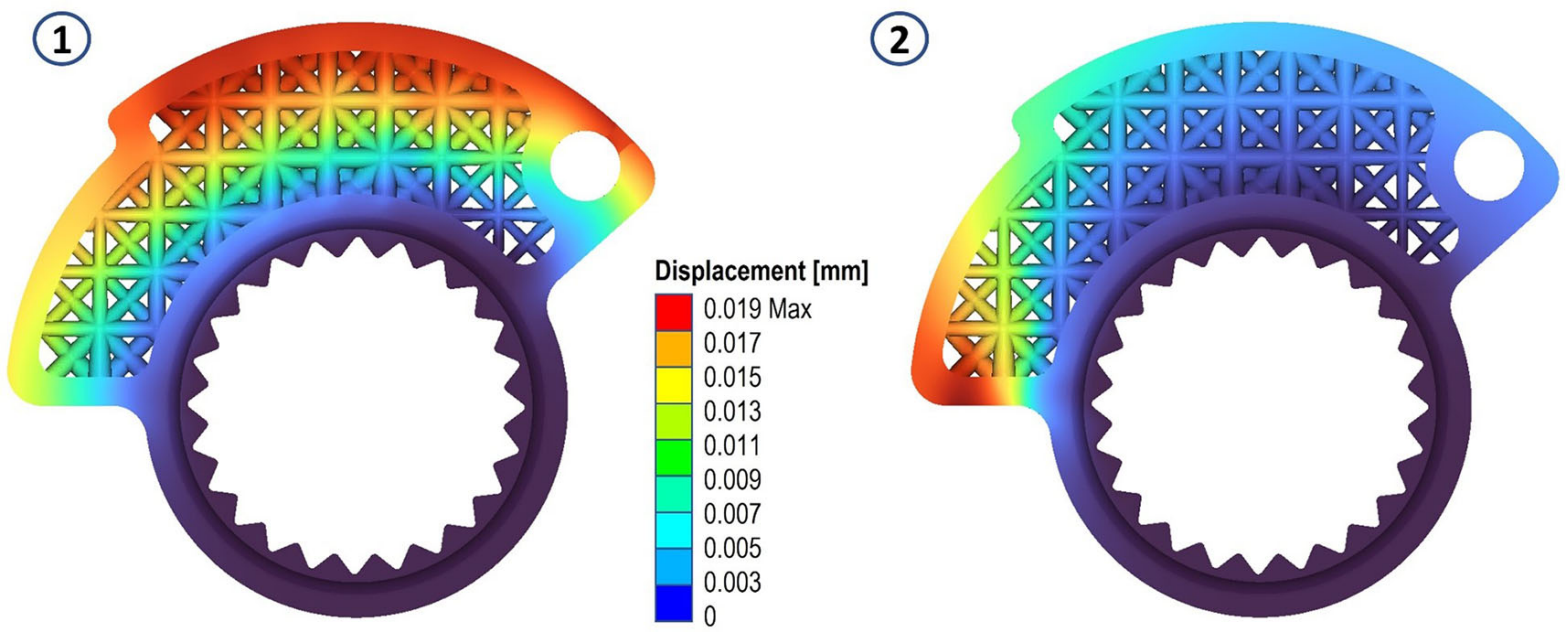
Displacements for the final structures, case 1 and 2.

**Figure 10.**
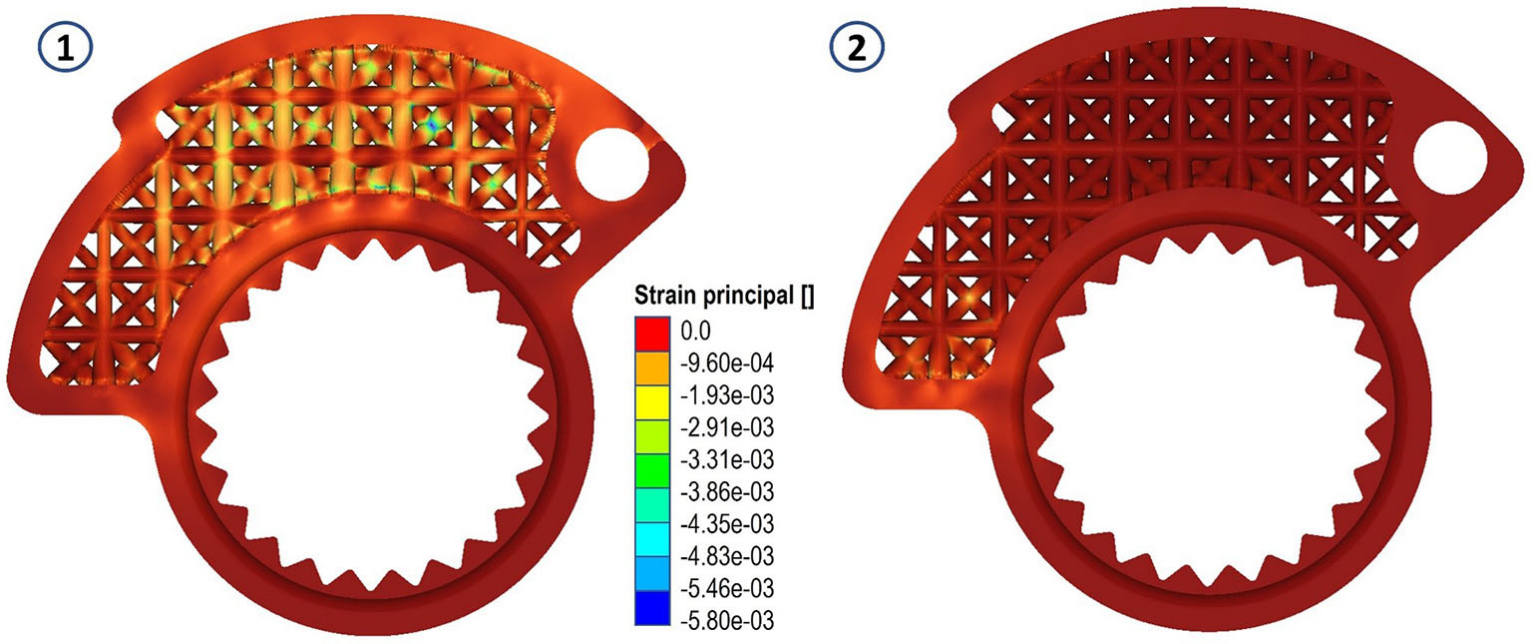
Strain evaluated for the final structures, case 1 and 2.

Areas with von Mises stress peaks of about 14 MPa were recorded in both cases. In both cases the peaks are compressive stresses, not particularly problematic for the life of the component. This can be seen by looking at the following figures ( Figure 8, Figure 9 and Figure 10).

The final simulation results on the component show satisfactory behaviour’s that are in accordance with the design characteristics. We then proceeded with the export of the mesh in STL format to realize 3D printing of the component. The number of nodes in the exported mesh was 1087431. The need for a particularly dense mesh, wanting even more than that used for the structural verification of the component, is related to the fact that only with a mesh composed of many elements is possible a definition of the geometry of the component at high resolution ( Bacciaglia *et al.*, 2021).

### Printing strategy and settings

The printer is an EPAX E10 4K mono using MSLA technology. The resin for the models is an EPAX hard and Tough clear that has the mechanical properties described in Table 1, stated by the manufacturer.

The slicer used to prepare the model for printing is Lychee, version 1.7. It was decided to print the cam vertically, cause this position allows to keep good resolution and avoids warping outside the symmetry plane. The supports, placed manually, perform the triple function of supporting any “island” of material not connected to the rest of the part in a specific layer; preventing deformation due to the presence of overhangs or material shrinkage and pulling the part from the FEP layer with sufficient force. To compensate the shrinkage both in the printing and in the final curing phase a 101% scaling was added to the entire model, directly in the slicer.

Printing settings, according to the manufacturer specification, are set as shown in the table below ( Table 2). The high exposure value of the base layers (24 s) ensures the best possible adhesion to the aluminium printing platform. Normal layers, on the other hand, have a shorter exposure time (2.5 s), thus ensuring maximum detail resolution and avoiding overexposure that would lead to deformation and failure to meet prescribed dimensional tolerances. Slicing of the component was done in “.ctb (v4)” format, which allows vertical movement of the head in two different speeds. A low speed is therefore maintained in section 1, the one closest to the resin container, both in the ascent and approach phases. In contrast, the speed in section 2 is much higher. This makes it possible to avoid breakage or detachment of the part from the build plate in the phase of detachment from the FEP, and then to increase the speed when detachment is complete.

**Table 2.** Printing settings for the manufacturing process.

Parameters	Value	Unit
**Burn in layers**		
Number of layers	4	/
Exposure time	24	s
Lift distance (1)	4	mm
Lift distance (2)	3	mm
Retraction distance (2)	4	mm
Retraction distance (1)	3	mm
Lift speed (1)	50	mm/min
Lift speed (2)	150	mm/min
Retract speed (2)	150	mm/min
Retraction speed (1)	50	mm/min
**Normal layers**		
Exposure time	2.5	s
Lift distance (1)	4	mm
Lift distance (2)	3	mm
Retraction distance (2)	4	mm
Retraction distance (1)	3	mm
Lift speed (1)	50	mm/min
Lift speed (2)	150	mm/min
Retract speed (2)	150	mm/min
Retraction speed (1)	50	mm/min

After printing, the component is washed in IPA (isopropyl alcohol) through an ultrasonic cleaner. This allows the removal of any excess resin trapped in the component or inside the lattice geometry. The component underwent a 20-minute ultrasonic cleaning cycle, time suggested by the resin producer. Next, a manually support removal step was performed with the use of a little cutter. A careful visual inspection was performed in order to assess macro-defects such as failure of some parts of the structure, delamination or breakage occurring after the removal of the supports. The final step is the curing process to complete the polymerization of the part.

**Figure 11.**
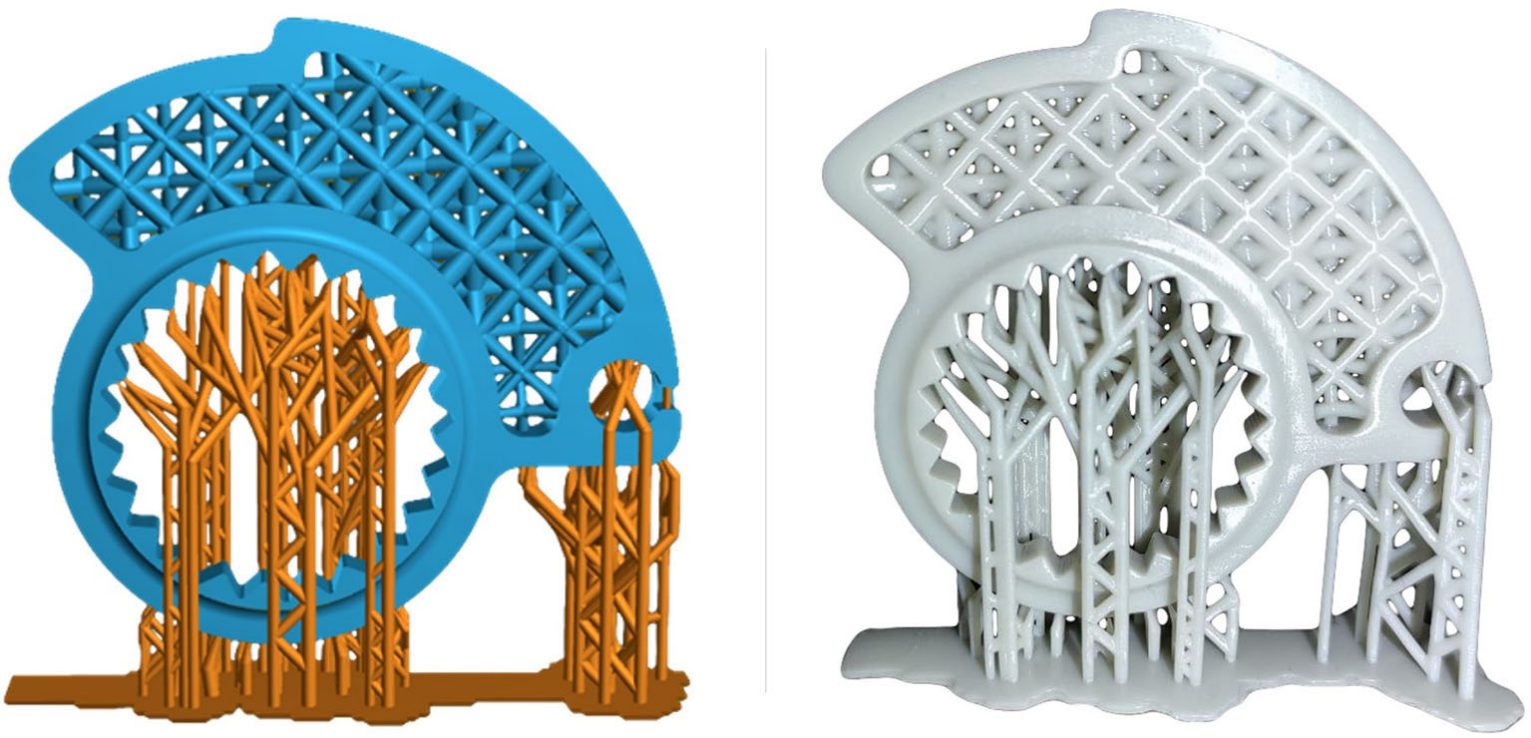
Detail of the component and the supports needed for printing, on the left, and the final part printed in resin with supports still to be removed, on the right.

Curing was performed using the XYZ printing 180 Multicure station. Following the manufacturer’s directions, a curing time of 15 minutes was set with UV lights ranging between 385-405 nm at 120 W. After the curing phase, an additional visual inspection phase of the component is performed. The inspection is performed to check for macroscopic cracks or visible deformation occurred after the curing phase. Then, the components were 3D scanned ( Figure 11) to assess if the dimensional tolerances of the component are within the needed for specific application.

**Figure 12.**
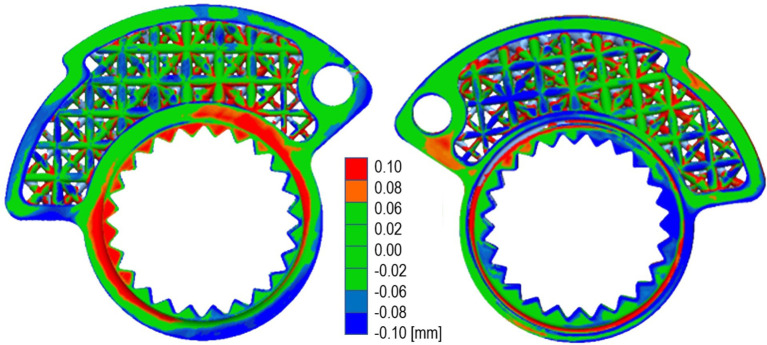
Quality control on the final resin prototype.

By evaluating the deviations between the CD model and the scanned prototype surfaces, the validation of the latter was made possible. Alignment between the CAD model and the scan mesh was performed in a way that ensured minimal overall error on the gap between the two surfaces. In the areas characterized by larger deviations, the gap between the CAD model is still less than 0.1 mm, as seen in Figure 12.

**Figure 13.**
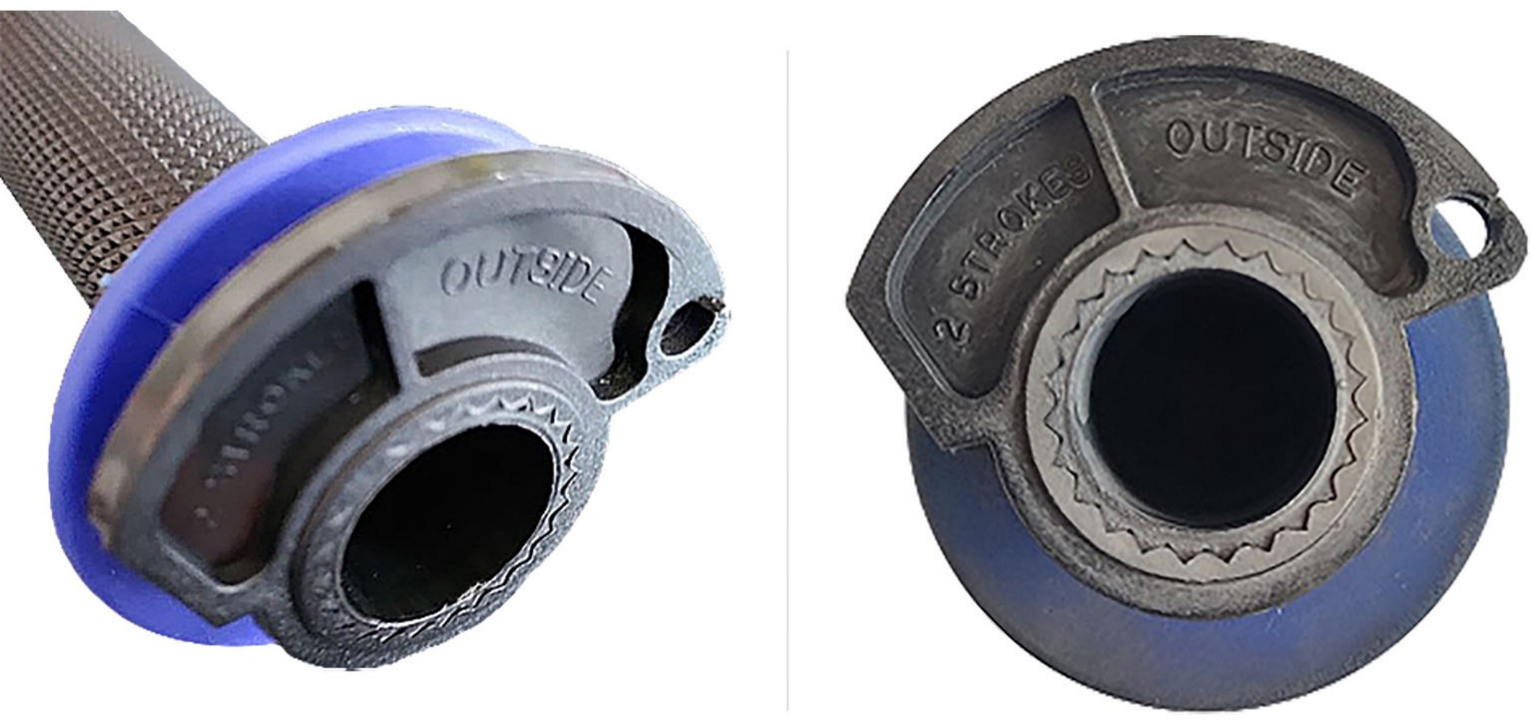
Original cam.

**Figure 14.**
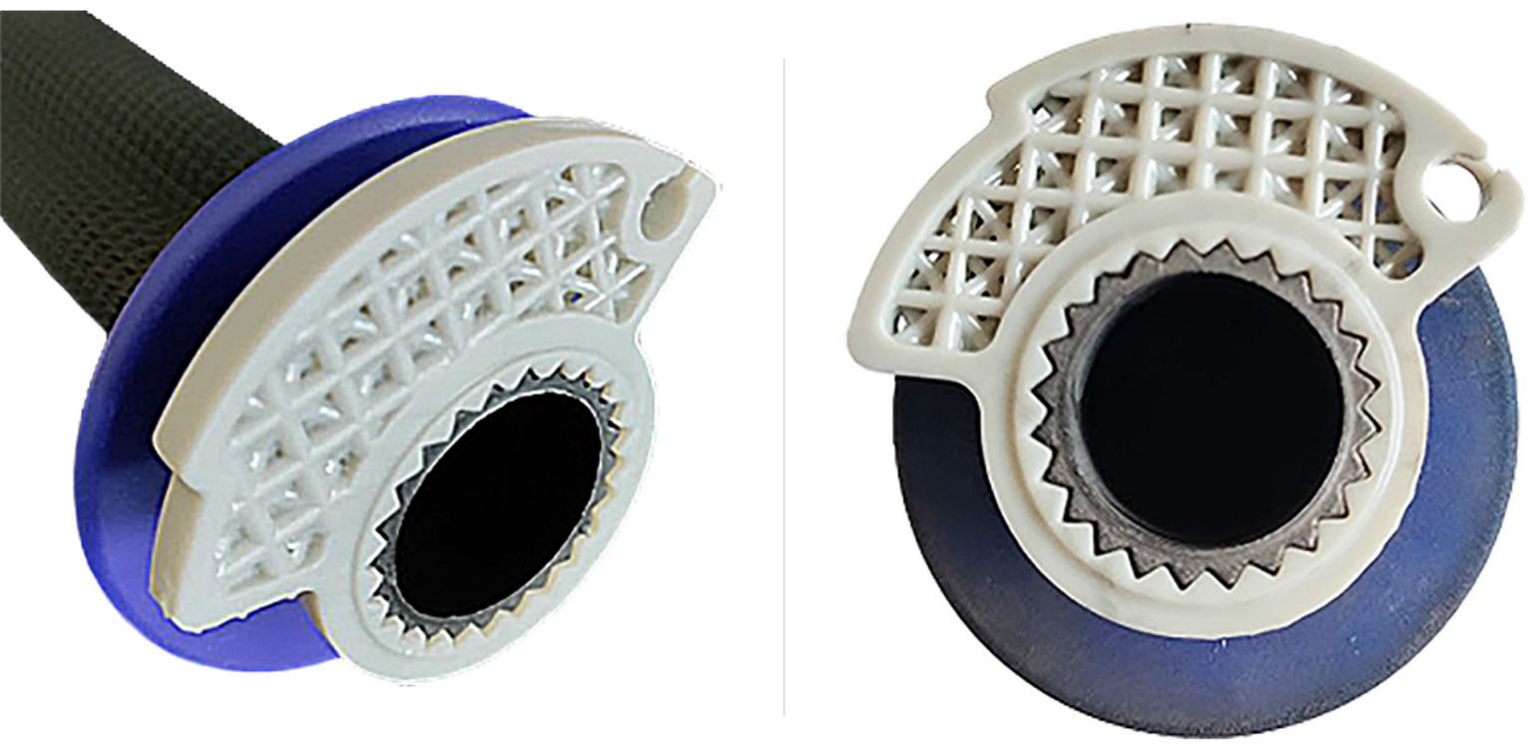
Mounted part on Progrip ^®^ mod.: 708 grip.

The CAM were then assembled on the Progrip ^®^ grip and subsequently used as can be seen from the following figures ( Figure 13 and Figure 14).

## Conclusions

Thanks to the advantages offered by Additive Manufacturing and, in particular, by 3D printing combined with the use of “lattice” structures, it has been possible to create an optimized functional component. This component is able to guarantee the maximum feeling between motorbike and rider and to allow the maximum integration between the two. A workflow was therefore proposed that can be optimally followed not only for the optimisation of the specific component of this article but for any component designed with latex structures and realised in resin. Within the workflow there are multiple control steps that allow defects to be immediately identified and traced back to a specific stage. This makes it possible to identify the origin of the defect and act accordingly.

Resin printing combined with new-generation resins has proven to be a mature technology for the production of functional components and not just prototypes. The final deformations of the object are small and within the imposed tolerances. It would be desirable in the future, especially in the case of components with higher tolerances, to be able to effectively simulate the printing process and thus predict the position of the substrates in order to minimize the deformation of the component and improve its final quality.

As far as “lattice” structures are concerned, these have been the subject of research as far back as the 1990s. Despite that they have only found a real possibility of application in the present day, thanks mainly to 3D printing technologies that allow great freedom in geometries. The possibility of applying “lattice” structures by combining them with a stress field or a deformation field and then modifying the thickness of the rafters that make up the cell according to a criterion greatly broadens the horizons towards increasingly optimized and high-performance structures.

## Data availability

All data underlying the results are available as part of the article and no additional source data are required.
