# Stress-Based Lattice Structure Design for a Motorbike Application

**DOI:** 10.12688/f1000research.125184.2

**Published:** 2023-11-29

**Authors:** Patrich Ferretti, Elena Fusari, Giulia Alessandri, Marco Freddi, Daniela Francia

**Affiliations:** 1 Department of Industrial Engineering, University of Bologna, Bologna, Italy, 40136, Italy

**Keywords:** MSLA, water washable resin, lattice structure, nTopology, Design for additive manufacturing, Stereolithography tooling, Shape optimization, CAD/CAM

## Abstract

**Background:**

The “drive by wire” mechanism for managing the throttle is not applied to every modern motorcycle, but it is often managed through a steel wire. Here, there is a cam on the throttle control. Its shape allows the throttle opening to be faster or slower and its angle of rotation, required for full opening, to be greater or less. The maximum angle a rider’s wrist can withstand depends on numerous musculoskeletal mobility factors, often limited by falls or surgery.

**Methods:**

Using a Progrip knob with interchangeable cams allows the customization of a special cam profile, to ensure the best engine response to throttle rotation and ergonomics for the rider. The use of FEA software and lattice structures, allows to realize a lightweight and efficient design, targeted for fabrication with additive manufacturing technologies.

**Results:**

The cam was manufactured by exploiting MSLA technology. Finally, a dimensional inspection procedure was performed before assembly. The main result is to have obtained a lighter and cheaper component than the original.

**Conclusions:**

This study has allowed the design of a mechanical component consisting of innovative shape, light weight, and ergonomics. Furthermore, it demonstrates the effectiveness in the use of lattice structures to enable weight optimization of a component while minimizing the increase in its compliance.

## Introduction

Presently, Additive Manufacturing (AM) stands as the sole technology capable of ensuring the cost-effective production of customized components. Leveraging the latest structural optimization software and lightweighting capabilities enables the design of these components. 3D printing presents a versatile opportunity to manufacture components using diverse materials, ranging from polymeric substances to metals. When discussing the design of components featuring “lattice” structures, additive manufacturing invariably emerges as the sole viable choice ( Pan *et al.*, 2020). Many recently developed geometries have the distinction of being weight-optimized through the use of special internal “lattice” structures ( Gibson, 1989; Tamburrino *et al.*, 2018; Tao & Leu, 2016). These are merely internal ramifications of the geometry that can provide good stiffness while leaving numerous gaps to minimize the weight of the final structure ( Messner, 2016). The primary advantage lies in maintaining an unchanged external geometry of the component. This factor holds significant importance, given that the component’s shape frequently serves as the principal design constraint. The internal patterns of these geometries can be composed of straightforward shapes, including hexahedra or tetrahedra cells, octets ( Deshpande *et al.*, 2001) or classical hexagonal honeycombs, or more complex surfaces governed by mathematical equations in 3D space ( du Plessis *et al.*, 2018). A classic example is the Gyroid structure ( Khaderi *et al.*, 2014), but many others exist, such as Neovius cells ( Khan & Abu Al-Rub, 2017), Lidinoid and Schwarz ( Shin *et al.*, 2012). These belong to “TPMS” (Triply Periodic Minimal Surface) cells category, much deployed in this design context ( Al-Ketan & Abu Al-Rub, 2019; Gandy *et al.*, 2001; Jung *et al.*, 2006; Savio *et al.*, 2019).

### MSLA (Mashed Stereolithography) Resin Printing

Stereolithography (SLA), a patented printing technology from the 1980s, employs a laser to fabricate components. The laser precisely targets the transparent bottom of a photosensitive surface within a reservoir filled with resin. This resin, responsive to ultraviolet light, undergoes curing and solidification solely in the specific regions irradiated by the laser, resulting in the formation of a material layer. Typically, the resins utilized in MSLA technology consist of thermoset polymers, renowned for their favorable mechanical properties and widespread industrial applicability ( Croccolo *et al.*, 2019). The process is repeated until the component is created. This process offers numerous advantages stemming from the superior isotropy of the printed component, coupled with exceptional surface finish and intricate detailing. Currently, SLA technology is undergoing various advancements geared toward significantly reducing costs and enhancing accessibility to the public, all while retaining its inherent benefits. One such advancement is ‘MSLA’ (Masked Stereolithography), which notably improves upon traditional ‘SLA’ technology in terms of part production speed. MSLA utilizes an LCD screen within the printer, featuring pixels whose quantity directly influences the resolution of the manufactured part. The screen functions as a mask for ultraviolet light, creating the 2D layer drawing to be printed by selectively activating or deactivating pixels. This simultaneous photo-polymerization of each point within a layer eliminates the necessity for multiple laser paths, as seen in SLA. Resin-based printing technologies excel in realizing lattice structures, effectively constructing the beams composing the lattice and capturing intricate details, even at a microscale.

### Customization and 3D printed parts

The need of customised components is of great interest in numerous fields, ranging from biomedical ( Frizziero, Santi, Leon-Cardenas, Donnici, Liverani, Papaleo, *et al.*, 2021a, 2021b) to mechanical engineering especially the automotive field. For several years now there have been dedicated departments within the companies themselves to meet specific customer requirements ( Fantini *et al.*, 2016; Zhang *et al.*, 2021). Prior to the rise of Additive Manufacturing, the production of a singular mechanical component relied on one or multiple “traditional” technologies, which remain prevalent in today’s industry. This process often incurred exceedingly high and, at times, unmanageable costs, as these expenses were not rationalized by the production of a solitary component. In particular, the level of customisation that can be achieved through the use of 3D printing is unattainable by any other technologies, as well demonstrated in literature ( Ibhadode, 2023). This makes the additive manufacturing extremely competitive, both in terms of cost and of the complexity of the geometries that can be produced. Many examples in the literature demonstrate how additive manufacturing is the winning choice. The applications go from the aerospace sector ( Moon *et al.*, 2015; Totaro & Gürdal, 2009; Vasiliev & Razin, 2006; Zhu *et al.*, 2015) to the medical-health sector cited, from the automotive sector ( Bacciaglia *et al.*, 2020a; Yin *et al.*, 2018) to the entertainment sector ( Bacciaglia *et al.*, 2020b).

## Part modelling process and lattice creation

**Figure 1.**
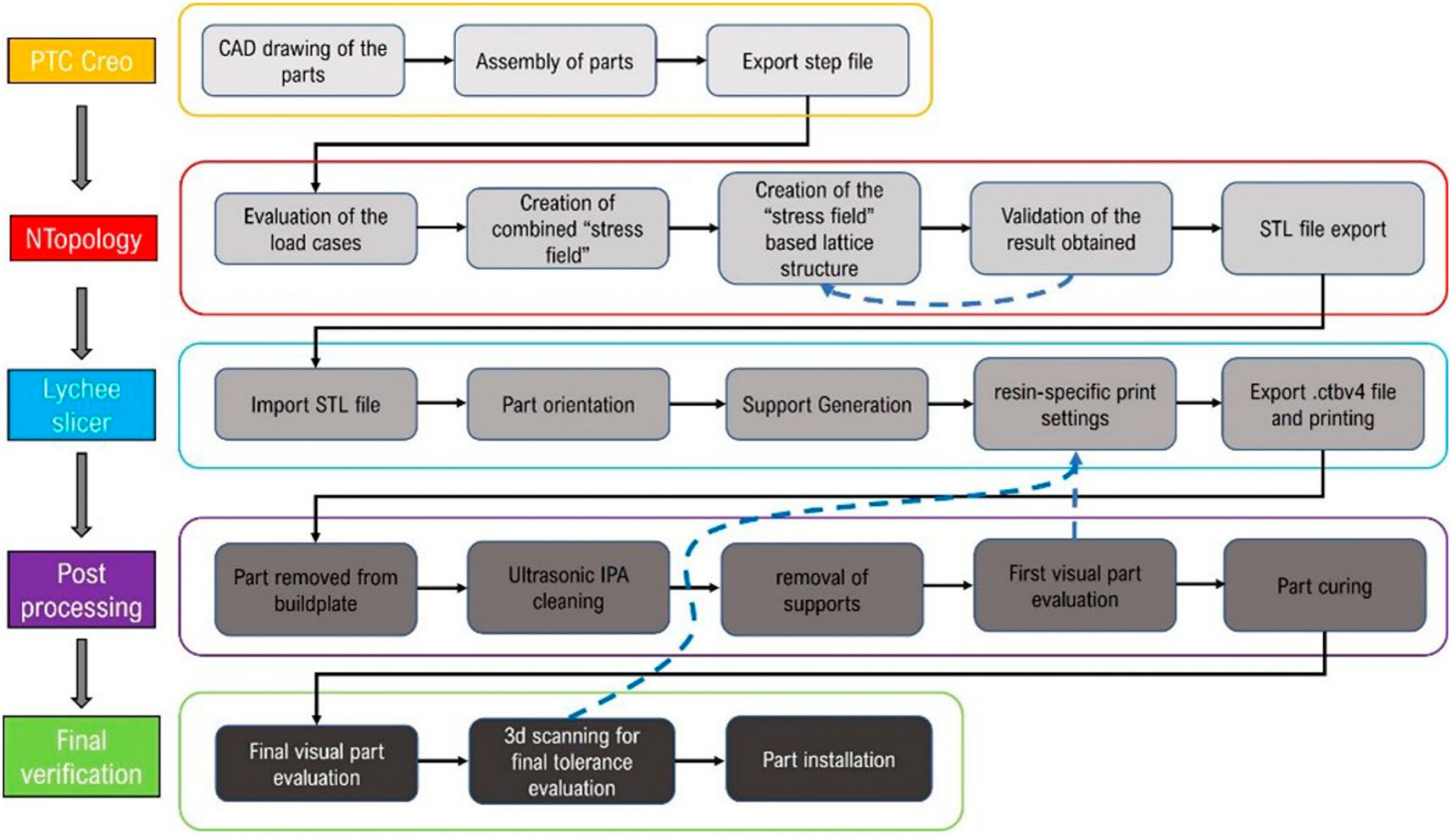
Project’s workflow.

The design of the components is conditioned by various factors: the production process adopted (resin printing); the perfect intercompatibility with respect to the Progrip mod.708 grip; the standard gas control scroll and finally the geometry required by the rider. The workflow followed is shown in Figure 1.

**Figure 2.**
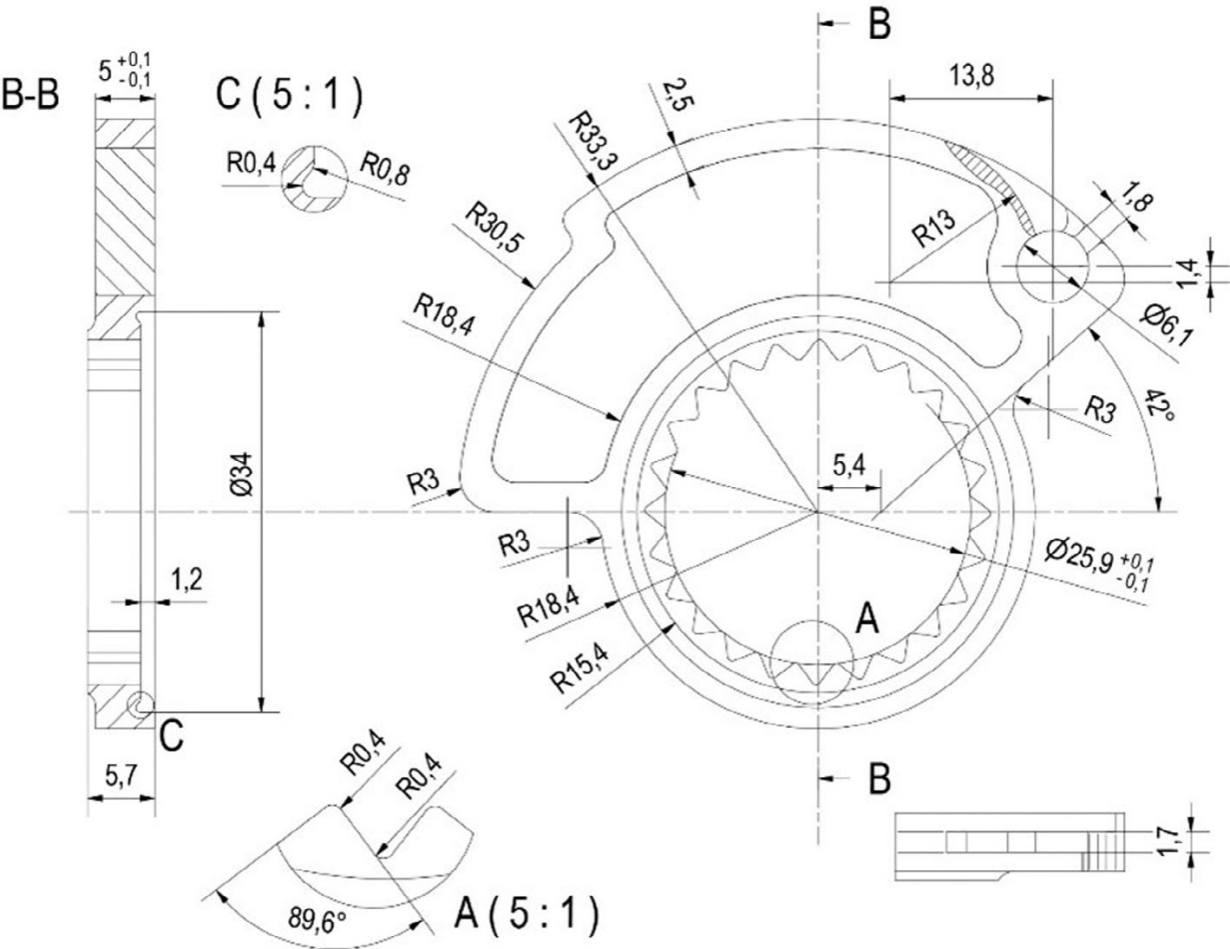
Mechanical drawing of the original cam.

The starting point is the CAD creation of an initial cam model that had similar overall dimensions to those provided in the Progrip kit, but with a custom profile. A quick action throttle cable, commonly referred to as “quick action cam,” is an accessory used in motorcycling to enhance the responsiveness of the throttle control, typically for performance improvements. The quick action throttle is primarily composed of a specialized throttle cable that is shorter in length compared to the stock throttle cable. This cable connects the throttle grip on the handlebar to the carburetor or fuel injection system. The shortened cable means that even a slight twist of the throttle grip results in a wider opening of the throttle body or fuel injector. This immediate response of the throttle provides a quick power increase when the rider twists the grip. The external profile of the cam possesses a slightly larger diameter compared to the cam provided by Progrip, enabling a full opening of the throttle valve within less than a 90° rotation of the grip lever. The default cam accompanying the Progrip knob demands an angle exceeding 90°. In our particular scenario, constrained by limited wrist mobility, this standard cam impedes the rider from fully extending the throttle. The original cam design is presented in Figure 2. This component is manufactured using Polyamide/Nylon PA6 and PA66.

**Figure 3.**
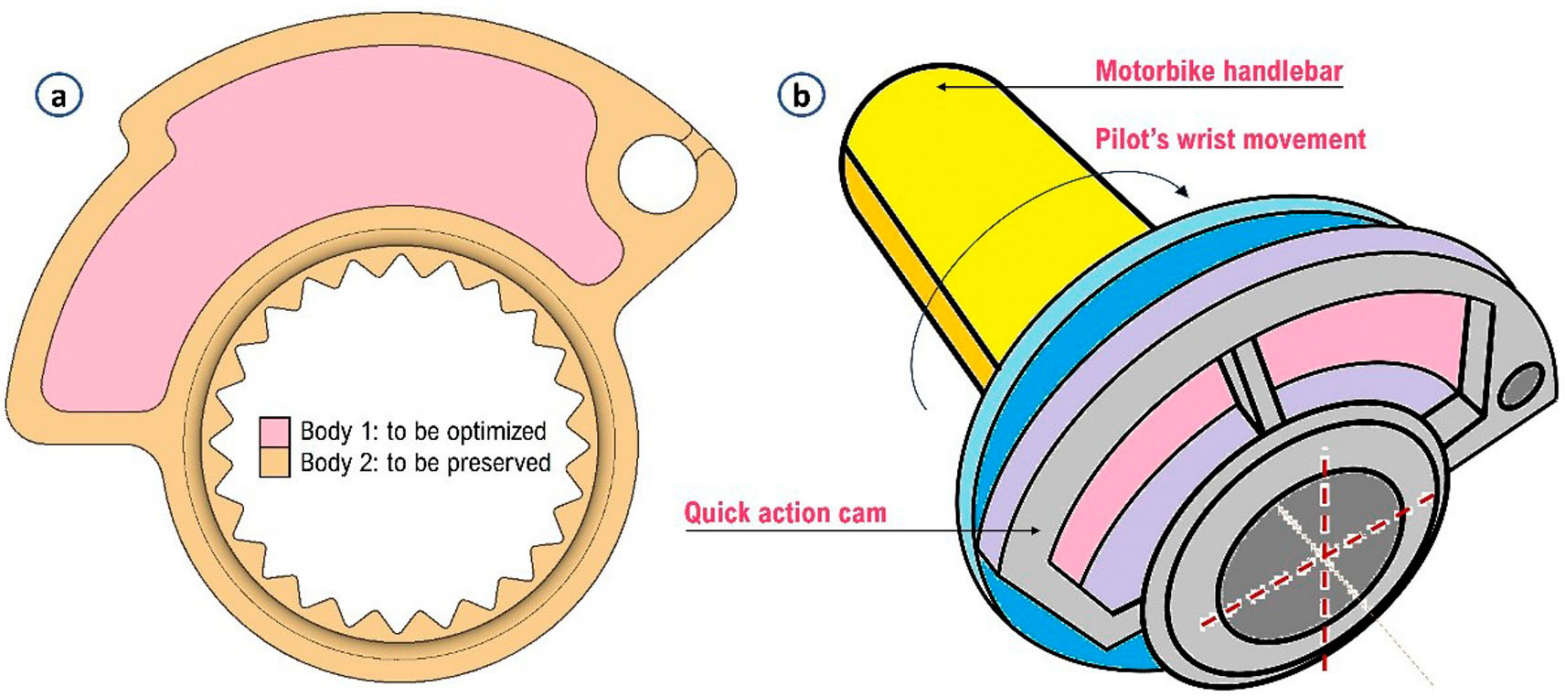
(a) Separation of the volume to be preserved from that for optimization; (b) Diagram of mounting/using the cam on a motorcycle handlebar.

The cam was then later divided into two separate bodies as can be seen from Figure 3. A body that is the unmodifiable geometry and the other one (central part) in which apply lattice optimization to minimize the material used during printing and, at the same time, ensure the maximum stiffness values and required performance. The two bodies were exported as an assembly file in STEP format. A clarifying assembly/usage diagram on the motorcycle handlebar is also presented in Figure 3.

**Figure 4.**
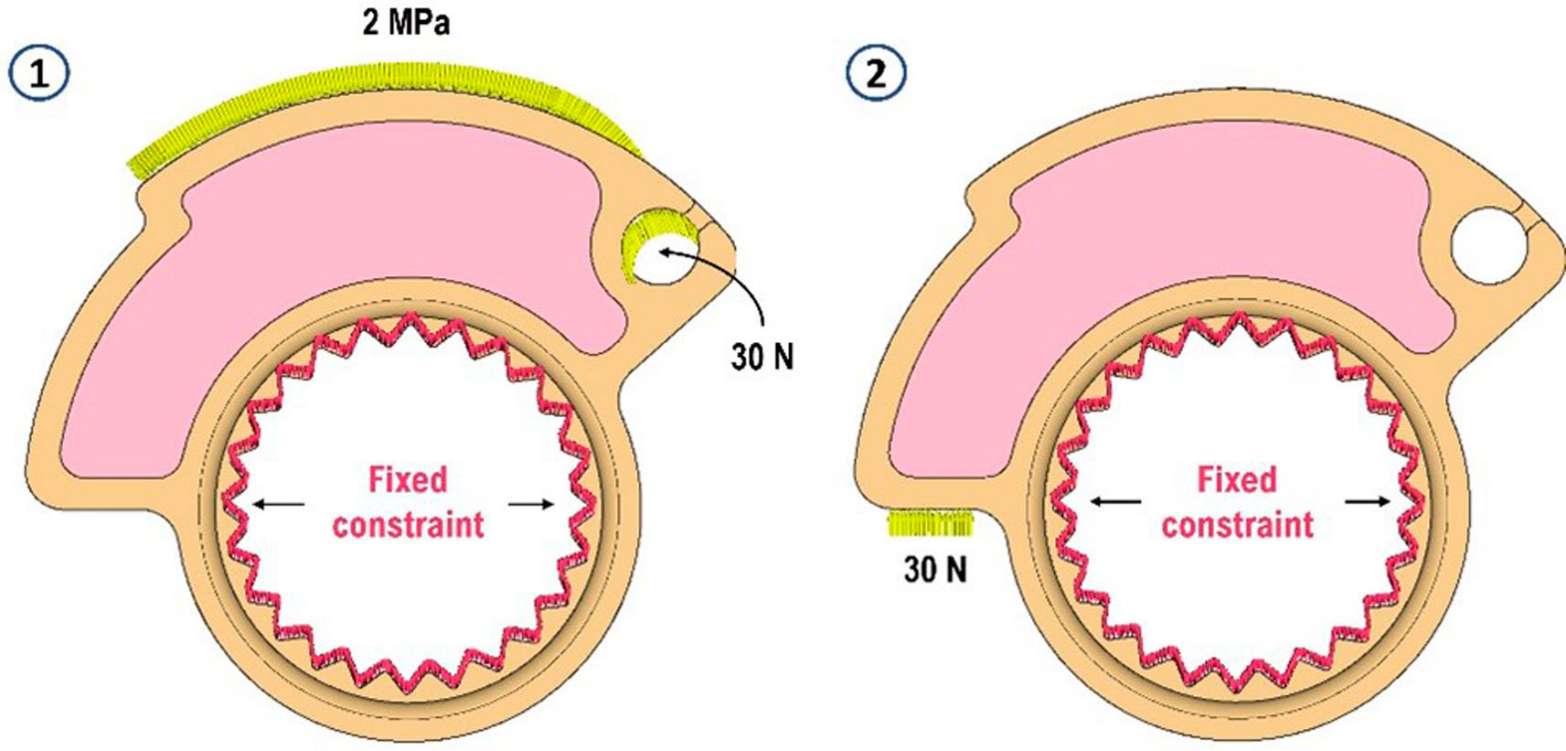
Load cases.

The geometry is then imported into nTop, which is a software dedicated to the design of lattice or topology-optimized components and subsequent structural verification. The component model is imported as an assembly, divided into two separate volumes. One is intended to remain unchanged at the end of the design process; the other is to be lightweighted with a lattice structure. The outer part of the throttle cam features a shape that was deemed non-optimizable. The edge of the cam around the central crown, utilized for mounting on the handlebar and suitable for transmitting the torque generated by the rider’s hand during acceleration, already possesses an optimized shape and thickness. The upper section of the cam is designed with a profile to achieve the necessary length when coupled with the constrained steel wire within the orifice, essential for adjustment purposes. The sole modifiable and reducible volume is highlighted in pink. The process leading to the final generation and export of a differentiated density lattice structure is characterized by five distinct steps as shown in Figure 1. Within the nTop software, two simulations were configured, sharing a fixed constraint inside the cam while varying in applied loads. The initial simulation depicts the maximum throttle opening, where the throttle control is fully rotated, generating force through the return spring onto the cam via a steel wire. A force of 30 N was applied in the first case within the eyelet, acting tangentially to the termination of the fixed steel wire on the cam. To replicate the contact pressure between the cam and the steel wire, 2 MPa of pressure was evenly distributed across an area resembling the wire’s dimensions. In the second load case, 30 N of force normal to the contact surface of the accelerator nut was applied to simulate the uncontrolled release of the gas grip, resulting in the impact of a specific part of the cam as an endstop against the throttle scroll. The two load cases can be seen in Figure 4. For the mesh, 0.5 mm maximum allowable length per tetrahedral element was imposed, and 0.01 mm maximum allowable gap between the actual shape of the component and the mesh. A growth rate of 2 was also imposed. The choice of second-order finite elements was made to ensure better accuracy in results at the expense of longer simulation times. In this case, the choice is most suitable when dealing with printed materials (resin) and particularly thin internal structures. These conditions result in the generation of 973431 elements and 200049 nodes. The contact between the two volumes was defined and set as a perfect bonding (Structural Bonded Contact in nTop). Isotropic properties were assigned for the material as given below: Young’s modulus of 1930 MPa (average value compared with the properties stated in Table 1) and Poisson’s coefficient of 0.38. A material exhibiting linear-elastic behavior was initially assumed, a presumption that was subsequently validated by the minimal deformations observed in the component due to the applied loads and operational conditions. Both structural simulations, utilizing identical meshes, were executed, and the resultant displacement and stress scalar fields were stored for future analysis.

**Table 1.** Mechanical properties of the resin used for printing the prototype.

Parameters	Value	Unit
Viscosity	150-300	MPa*s
Density	1.05-1.25	g/cm ^3^
Flexural Modulus	1682-2175	MPa
Flexural Strength	40-70	MPa
Heat Distortion Temperature	80	C°
Linear Shrinkage Rate %	1.05-1.35	/
Tensile Strength	30-48	MPa
Tensile Modulus	1779-2385	MPa
Elongation at Break	11-20	%
Poisson Ratio	0.38	/
Harness (Shore D)	70-80	/
Density after cured	1.09-1.18	g/cm ^3^
Notched impact Strength	41-48	J/m

**Figure 5.**
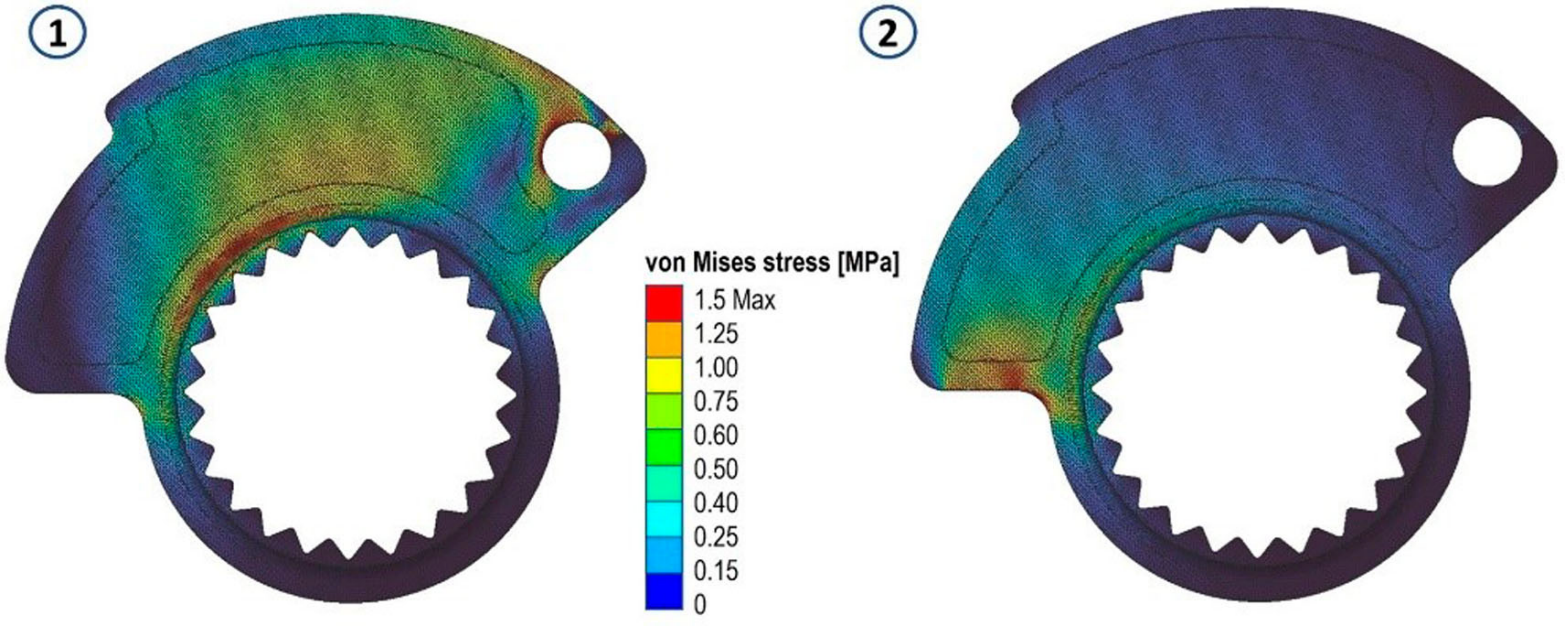
Von Mises stress evaluated for the two analyses.

**Figure 6.**
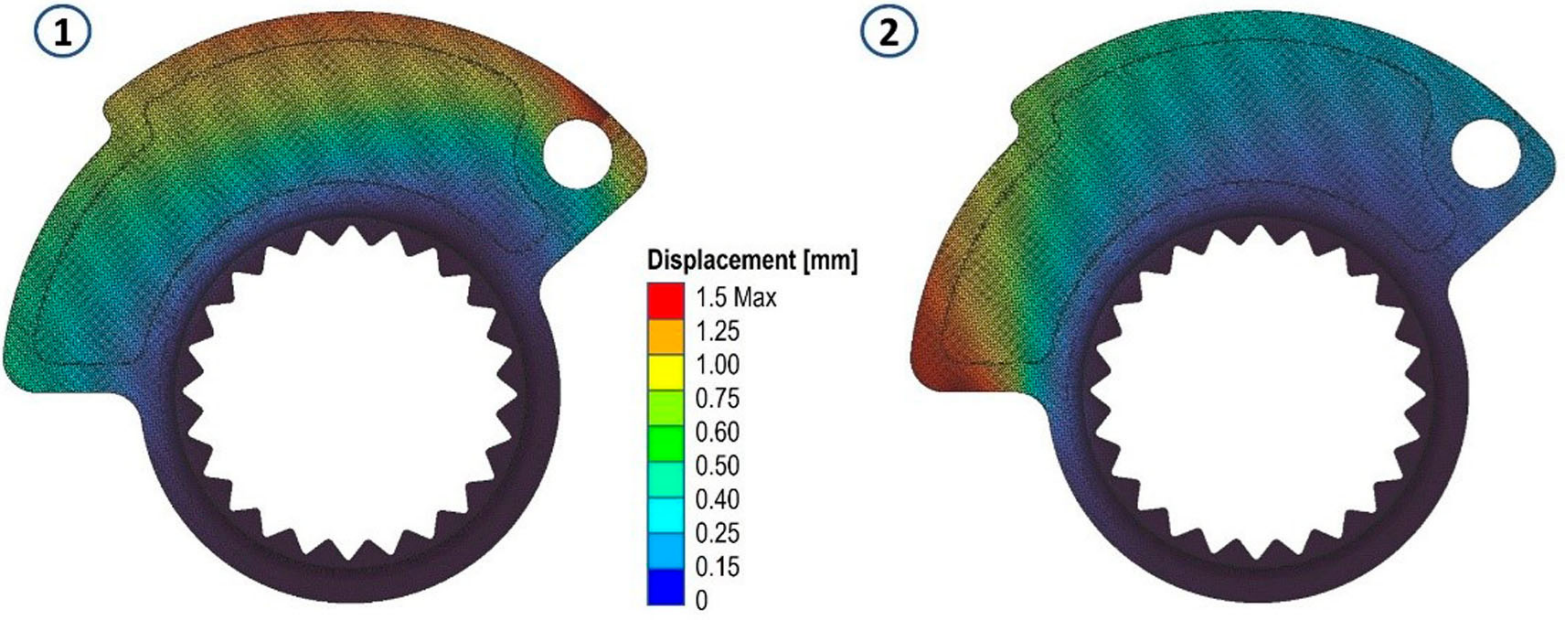
Map of local material displacements (in mm) analyzed in the first loading case and in the second both on the original cam.

The results in terms of stress and displacements are shown in Figure 5 and Figure 6. Then, a scalar field (Field from Point Map) has been defined as a function from R3 to R1, taking as input the spatial position (x, y, z) of a node in the component mesh and returns as output the von Mises stress value evaluated at that node.

In handling two distinct load cases and their respective stress fields, our approach involved defining an overall stress field. This aggregate field assigns each node within the domain the highest stress value obtained from a comparison of the values at that specific node in the two fields. This method ensures the consideration of any stress peaks present in either load case. Utilizing an average of the values from the two fields would have precluded this outcome.

**Figure 7.**
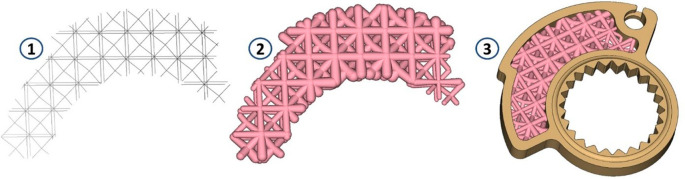
Sequence of steps constructed from the definition of the lattice grid (1) to its thickening based on the stress field (2), to the creation of the final part ready to be validated (3).

To develop a final design capable of accommodating the density variation directly linked to stress variation within the component, determining the cell type and truss dimensions becomes imperative. The choice was to use the Tet-oct vertex centroid type, with a cell size equal to 7 mm. The selection of this cell type primarily stems from its capability to provide medium to high material density, ensuring minimized regions devoid of material within the volume undergoing shape modifications in the component. Additionally, its compatibility with resin material for printing contributes to its ease of manufacturability. The lattice thus defined is trimmed so that it remains included in the volume intended for lattice optimization (1); then, we proceed by removing the “floating beams,” elements disconnected from the remaining lattice defined in that volume. At that point, we proceed by defining the thickness variability (Thicken Lattice) according to the stress field. In the case under consideration, the minimum stress value was assigned a thickness of 0.8 mm, and the maximum value of 2.5 mm. These values were defined following an optimization loop as can be seen by the blue arrow in Figure 1. The goal was to minimize the volume of material use, trying to contain stress and deformations at the same time. The value of 0.8 mm represents the minimum beam thickness achievable with this resin. This means that going below this thickness does not allow the realization of the truss beams. A useful representation for understanding these last design steps is offered in Figure 7.

Re-simulating the component’s behavior is crucial as material removal diminishes its overall stiffness. Additionally, the interconnections between trusses within the lattice zone may induce localized stress peaks. To validate the resulting structure, a new mesh configuration needs defining, with two possible approaches available. Firstly, employing a highly dense global mesh would meticulously capture the intricacies of each section within the variable-thickness lattice. Although this method demands greater computational time for meshing and simulation, it effectively captures localized effects.

Alternatively, for an isotropic material, a mesh featuring finite “beam” elements within each lattice segment is viable. While this approach forfeits the capacity to evaluate localized stresses, it ensures swift and accurate deformation predictions. However, it provides approximated values for internal stresses in the output. Despite this limitation, it serves as a valuable tool for swiftly evaluating results in designs primarily driven by stiffness considerations. In the present case, the first choice was made. The maximum element size of the new mesh was set to 0.1 mm, and a total of 1913956 elements corresponding to 427668 nodes were generated. The simulations of the same load cases illustrated above were performed.

**Figure 8.**
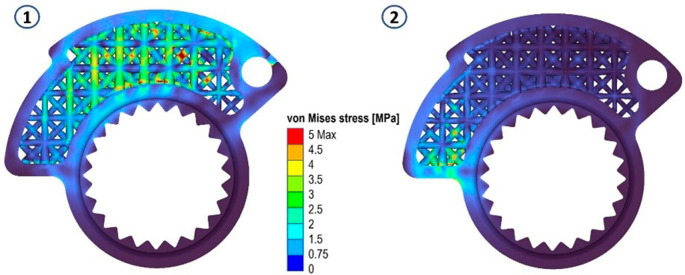
Von Mises stress for the final structures, case 1 and 2.

**Figure 9.**
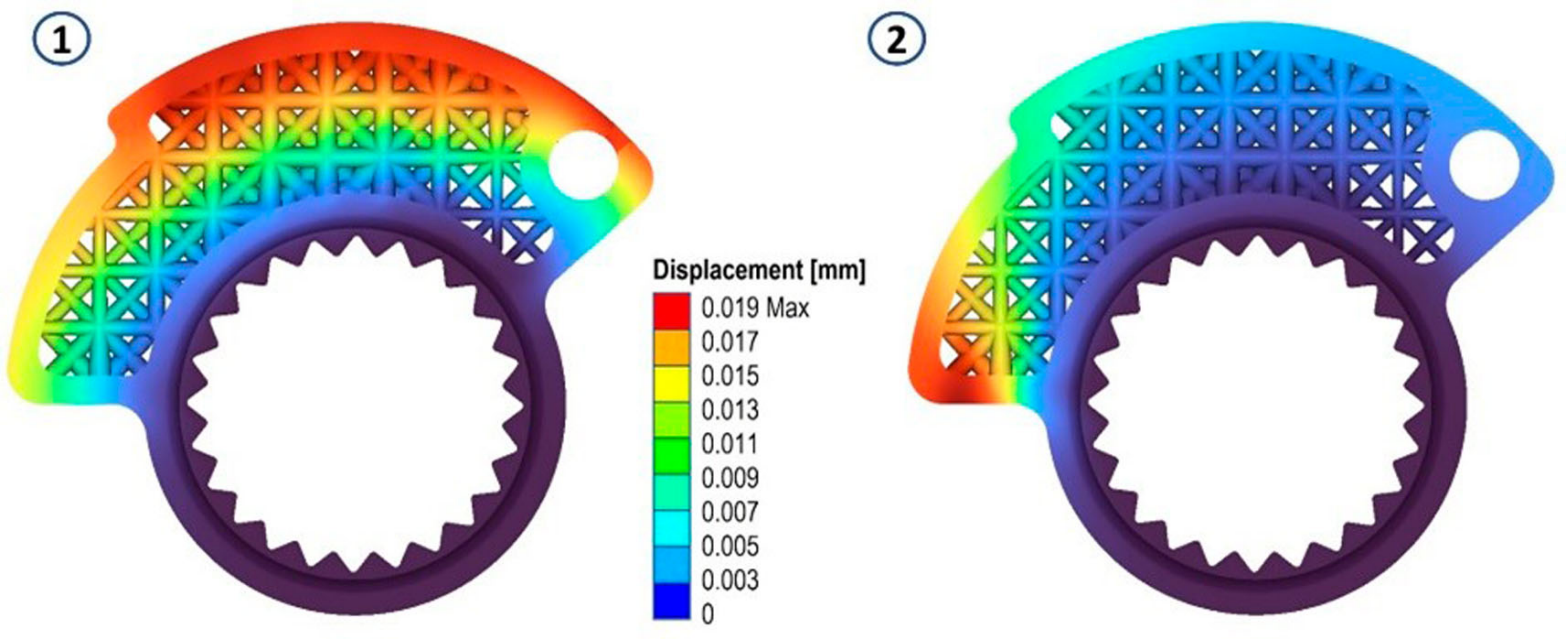
Map of local material displacements (in mm) analyzed in the first loading case and in the second on the modified cam.

**Figure 10.**
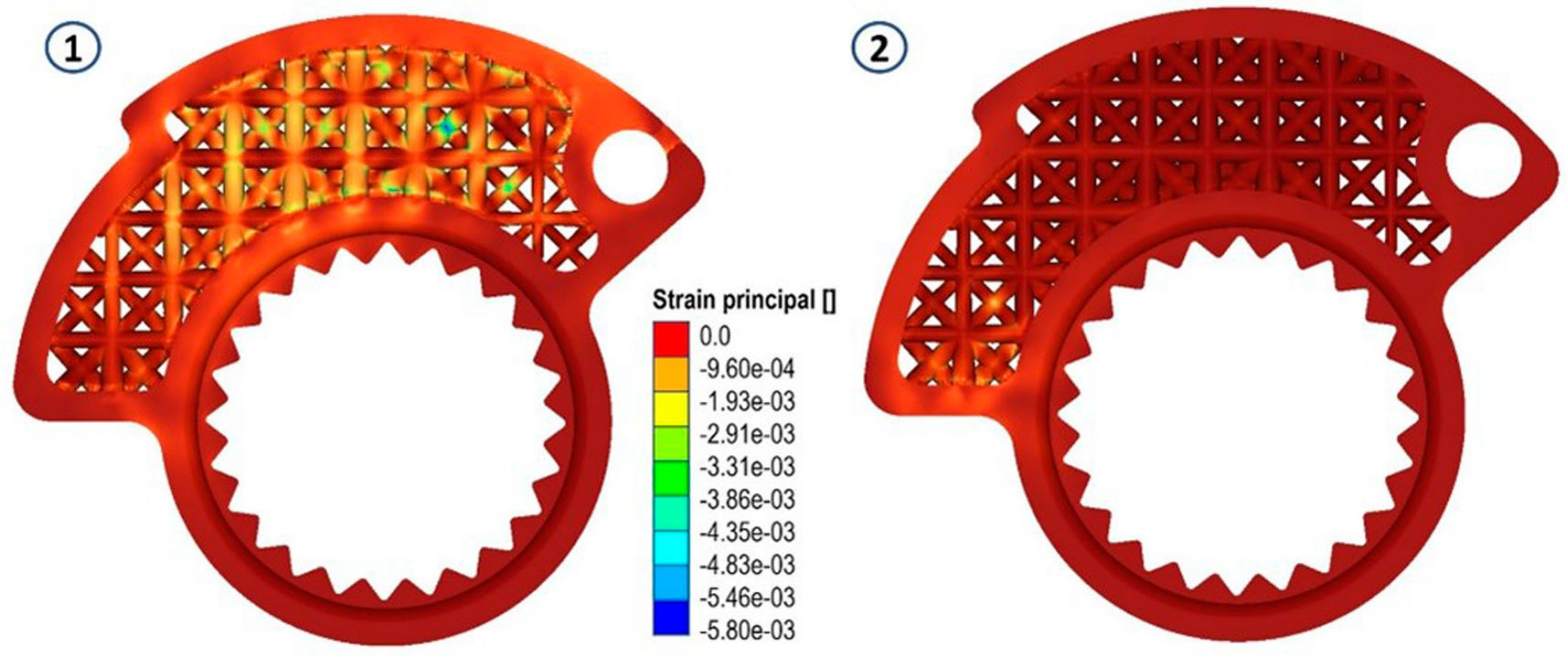
Maximum principal strain evaluated for the modiefied cam, case 1 and 2.

In both scenarios, zones registering von Mises stress peaks of approximately 14 MPa were identified. These peaks predominantly exhibit compressive stresses, which, in terms of the component’s lifespan, do not pose significant concerns. This can be seen by looking at the following figures ( Figure 8, Figure 9 and Figure 10).

The conclusive simulation results of the component exhibit behavior aligning with the intended design characteristics. Subsequently, we initiated the mesh export in STL format for the 3D printing of the component. The exported mesh contained a total of 1,087,431 nodes. The requirement for an exceptionally dense mesh, surpassing that employed for the structural validation of the component, is attributed to the necessity for a high-resolution definition of the component’s geometry. Such a dense mesh, comprised of numerous elements, is imperative for achieving detailed geometry resolution ( Bacciaglia *et al.*, 2021).

### Printing strategy and settings

The printer is an EPAX E10 4K mono using MSLA technology. The resin for the models is an EPAX hard and Tough clear that has the mechanical properties described in Table 1, stated by the manufacturer. The slicing software employed for preparing the model for printing is Lychee, version 1.7. Opting to print the cam in a vertical orientation was deliberate, as this position maintains superior resolution and mitigates warping issues beyond the symmetry plane. Manually placed supports serve a triple function: first, they support any disconnected “islands” of material within a specific layer; second, they prevent deformation caused by overhangs or material shrinkage; and third, they facilitate adequate detachment of the part from the FEP layer by applying sufficient force. To counteract shrinkage during both printing and the final curing phase, a 101% scaling was uniformly applied to the entire model directly within the slicer. This compensatory measure aims to address any potential dimensional alterations in the printed and cured component.

Printing settings, according to the manufacturer specification, are set as shown in the table below ( Table 2). The high exposure value of the base layers (24 s) ensures the best possible adhesion to the aluminium printing platform. Normal layers, on the other hand, have a shorter exposure time (2.5 s), thus ensuring maximum detail resolution and avoiding overexposure that would lead to deformation and failure to meet prescribed dimensional tolerances. Slicing of the component was done in “.ctb (v4)” format, which allows vertical movement of the head in two different speeds. A low speed is therefore maintained in section 1, the one closest to the resin container, both in the ascent and approach phases. In contrast, the speed in section 2 is much higher. This makes it possible to avoid breakage or detachment of the part from the build plate in the phase of detachment from the FEP, and then to increase the speed when detachment is complete.

**Table 2.** Printing settings for the manufacturing process.

Parameters	Value	Unit
**Burn in layers**		
Number of layers	4	/
Exposure time	24	s
Lift distance (1)	4	mm
Lift distance (2)	3	mm
Retraction distance (2)	4	mm
Retraction distance (1)	3	mm
Lift speed (1)	50	mm/min
Lift speed (2)	150	mm/min
Retract speed (2)	150	mm/min
Retraction speed (1)	50	mm/min
**Normal layers**		
Exposure time	2.5	s
Lift distance (1)	4	mm
Lift distance (2)	3	mm
Retraction distance (2)	4	mm
Retraction distance (1)	3	mm
Lift speed (1)	50	mm/min
Lift speed (2)	150	mm/min
Retract speed (2)	150	mm/min
Retraction speed (1)	50	mm/min

After printing, the component is washed in IPA (isopropyl alcohol) through an ultrasonic cleaner. This allows the removal of any excess resin trapped in the component or inside the lattice geometry. The component underwent a 20-minute ultrasonic cleaning cycle, time suggested by the resin producer. Next, a manually support removal step was performed with the use of a little cutter. A careful visual inspection was performed in order to assess macro-defects such as failure of some parts of the structure, delamination or breakage occurring after the removal of the supports. The final step is the curing process to complete the polymerization of the part.

**Figure 11.**
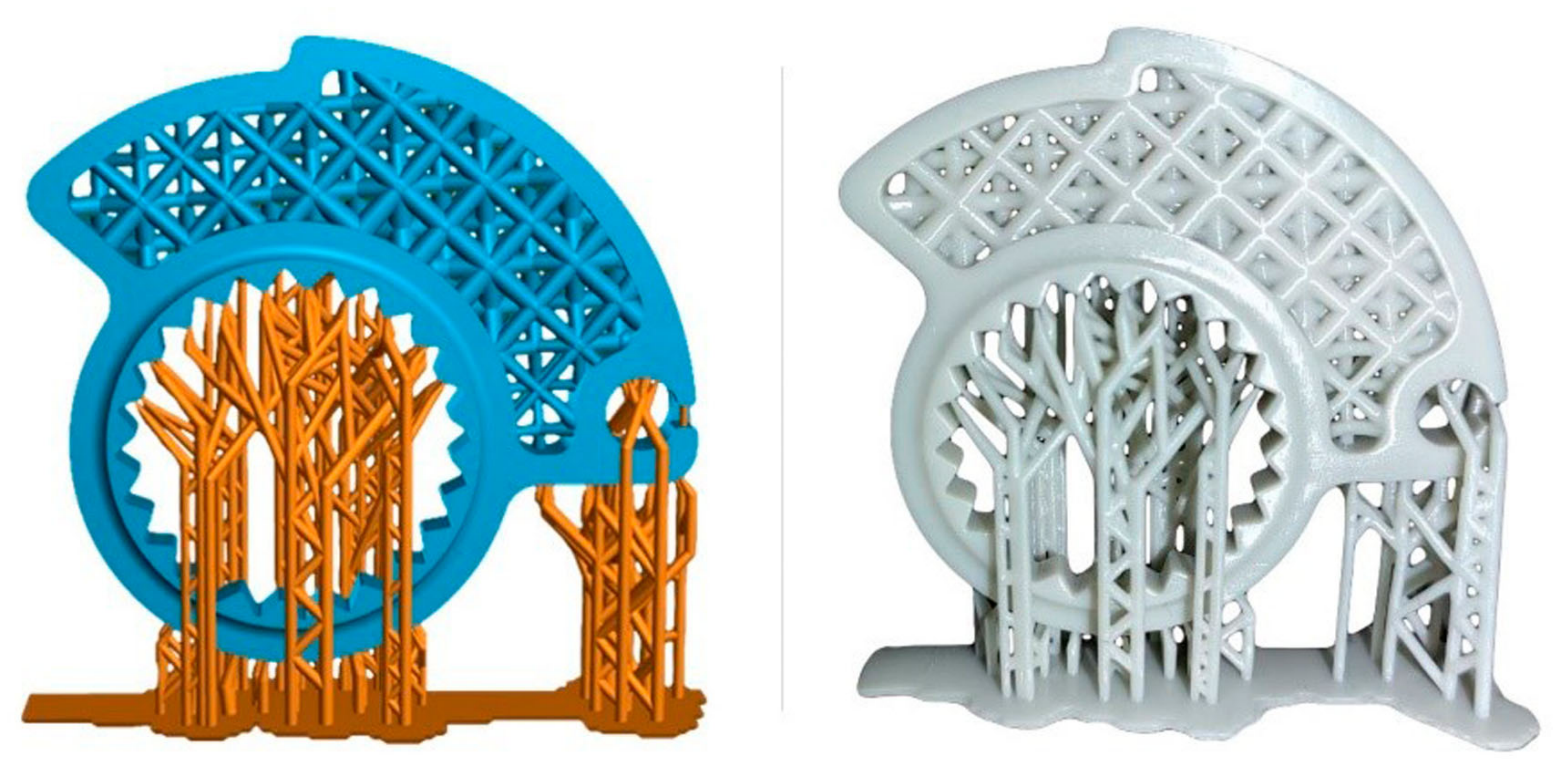
Detail of the component and the supports needed for printing, on the left, and the final part printed in resin with supports still to be removed, on the right.

Curing was performed using the XYZ printing 180 Multicure station. Following the manufacturer’s directions, a curing time of 15 minutes was set with UV lights ranging between 385-405 nm at 120 W. After the curing phase, an additional visual inspection phase of the component is performed. The inspection is performed to check for macroscopic cracks or visible deformation occurred after the curing phase. Then, the components were 3D scanned ( Figure 11) to assess if the dimensional tolerances of the component are within the needed for specific application.

**Figure 12.**
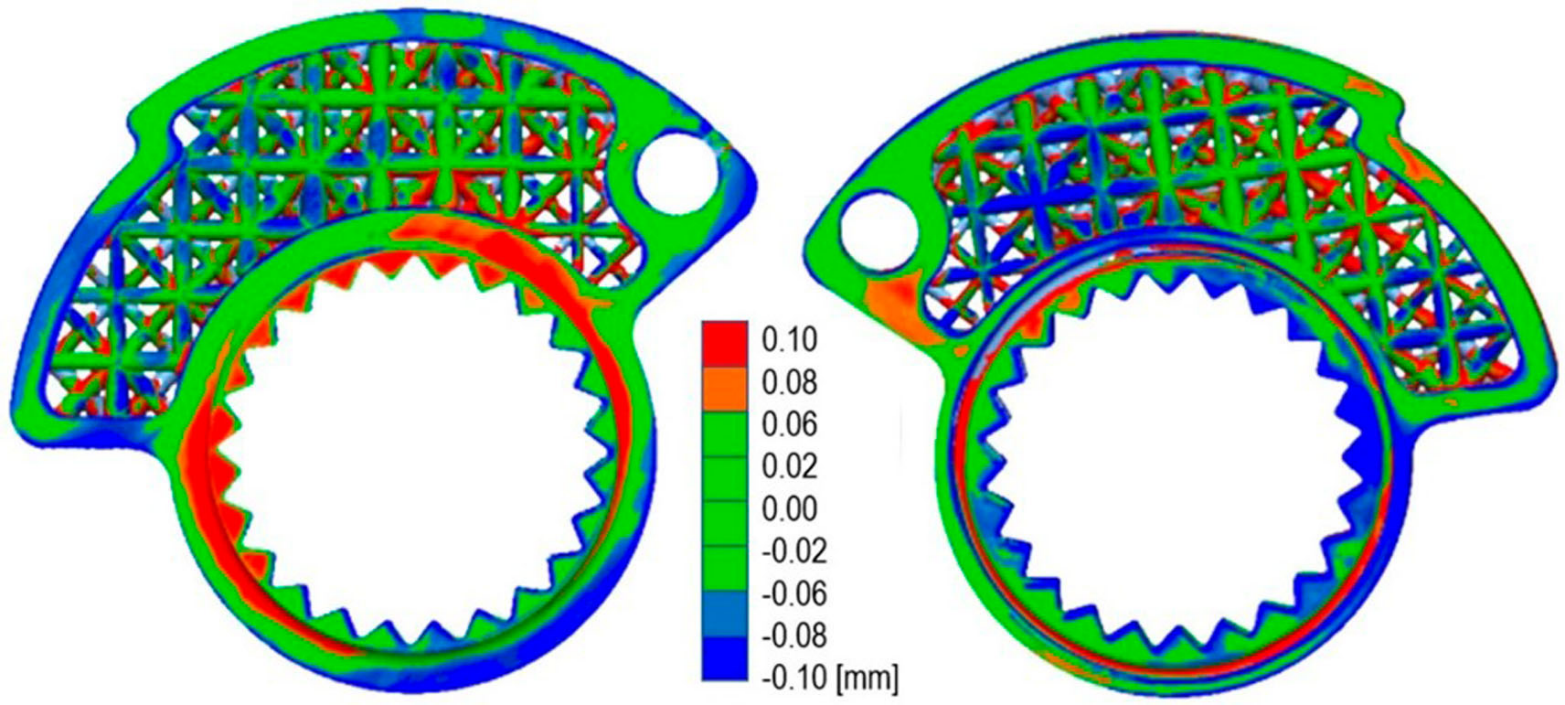
Quality control on the final resin prototype.

By evaluating the deviations between the Cad model and the scanned prototype surfaces, the validation of the latter was made possible. Alignment between the CAD model and the scan mesh was performed in a way that ensured minimal overall error on the gap between the two surfaces. In the areas characterized by larger deviations, the gap between the CAD model is still less than 0.1 mm, as seen in Figure 12. The FARO Quantum M ScanArm was utilized for the scanning process. Scanners of this kind do not require markers placed on or around the component’s surface. Manufacturers declare precision levels capable of ensuring an accuracy within 5 hundredths of a millimeter. This implies that from the scan, deviations from the actual surface of the component are expected to be locally within this specified range. The precision matches that of top-tier digital calipers, albeit restricted to measuring lengths, diameters, or simpler geometries.

**Figure 13.**
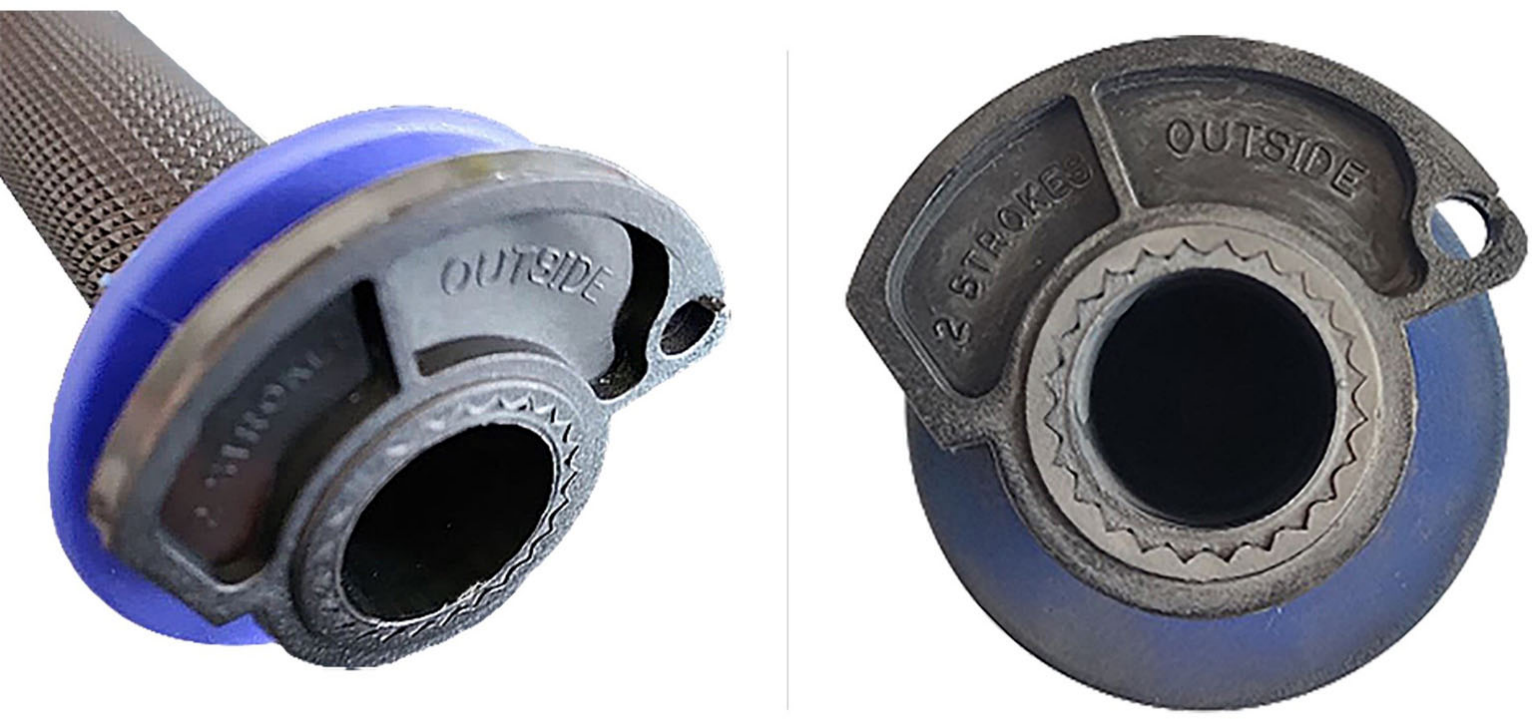
Original cam.

**Figure 14.**
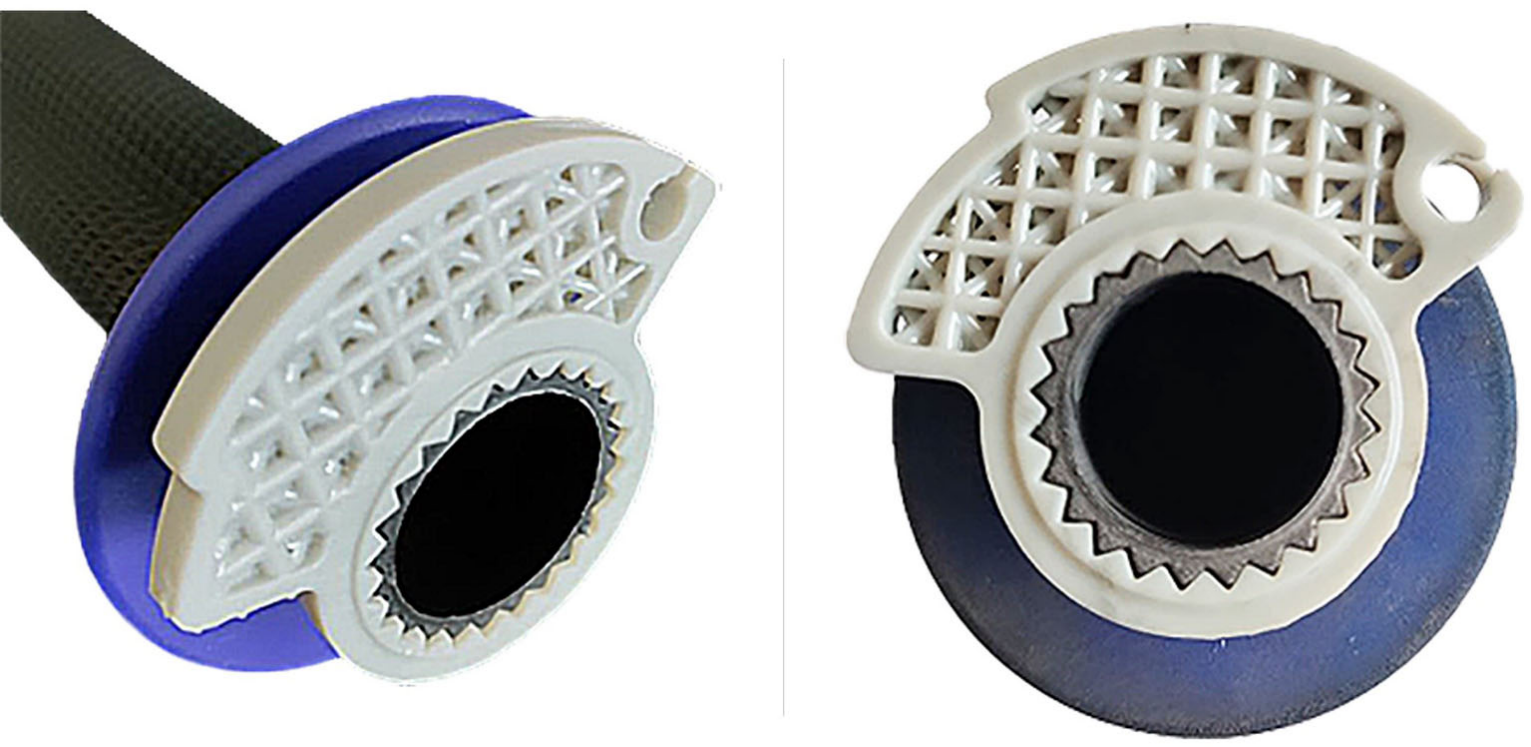
Mounted part on Progrip ^®^ mod. : 708 grip.

The CAM were then assembled on the Progrip ^®^ grip and subsequently used as can be seen from the following figures ( Figure 13 and Figure 14).

As can be seen by comparing the two figures, the cam end angle has been modified to prevent the rider from over-twisting the wrist. At the same time, the lightening of the component allows for better lightness and management of acceleration by the rider due to the reduced moment of inertia of the new model. This difference is slight and difficult to notice but is evident to the experienced hand of the pilot.

## Conclusions

Thanks to the advantages offered by Additive Manufacturing and, in particular, by 3D printing combined with the use of “lattice” structures, it has been possible to create an optimized functional component. This component is able to guarantee the maximum feeling between motorbike and rider and to allow the maximum integration between the two. A workflow was therefore proposed that can be optimally followed not only for the optimisation of the specific component of this article but for any component designed with latex structures and realised in resin. Within the workflow there are multiple control steps that allow defects to be immediately identified and traced back to a specific stage. This makes it possible to identify the origin of the defect and act accordingly.

Resin printing combined with new-generation resins has proven to be a mature technology for the production of functional components and not just prototypes. The final deformations of the object are small and within the imposed tolerances. It would be desirable in the future, especially in the case of components with higher tolerances, to be able to effectively simulate the printing process and thus predict the position of the substrates in order to minimize the deformation of the component and improve its final quality. Furthermore, a particularly straightforward type of internal infill was chosen to expedite the printing process (the application demanded short experimentation times within a sports and competitive context). Future research and experimentation activities in this field will focus on performance analyses of various structures to determine the optimal one in terms of stress resistance and printability.

As far as “lattice” structures are concerned, these have been the subject of research as far back as the 1990s. Despite that they have only found a real possibility of application in the present day, thanks mainly to 3D printing technologies that allow great freedom in geometries. The possibility of applying “lattice” structures by combining them with a stress field or a deformation field and then modifying the thickness of the rafters that make up the cell according to a criterion greatly broadens the horizons towards increasingly optimized and high-performance structures.

## Data availability

All data underlying the results are available as part of the article and no additional source data are required.
