# Supplementary material for: Stress-Based Lattice Structure Design for a Motorbike Application
Source: F1000Res. 2023 Nov 29;11:1162. Originally published 2022 Oct 11. [Version 2] doi: 10.12688/f1000research.125184.2 (PMC10799230; doi:10.12688/f1000research.125184.2)
Supplement: Peer review history

# Reviewer report: Reviewer response for version 2

**DOI:** 10.5256/f1000research.158715.r226783

**Published:** 2024-01-19

**Authors:** Craig M. Hamel

**Affiliations:** 1 Sandia National Laboratories, Albuquerque, Mexico

The authors have addressed my comments sufficiently.

Is the work clearly and accurately presented and does it cite the current literature?

Partly

If applicable, is the statistical analysis and its interpretation appropriate?

Not applicable

Are all the source data underlying the results available to ensure full reproducibility?

Yes

Is the study design appropriate and is the work technically sound?

Yes

Are the conclusions drawn adequately supported by the results?

Yes

Are sufficient details of methods and analysis provided to allow replication by others?

Partly

Reviewer Expertise:

Additive manufacturing, Constitutive modeling

I confirm that I have read this submission and believe that I have an appropriate level of expertise to confirm that it is of an acceptable scientific standard.

# Reviewer report: Reviewer response for version 2

**DOI:** 10.5256/f1000research.158715.r226782

**Published:** 2023-12-16

**Authors:** Osezua Ibhadode

**Affiliations:** 1 Mechanical Engineering, University of Alberta, Edmonton, Alberta, Canada

I believe substantial improvements have been made to the manuscript according to my recommendations; I approve it for publication.

Is the work clearly and accurately presented and does it cite the current literature?

Yes

If applicable, is the statistical analysis and its interpretation appropriate?

Not applicable

Are all the source data underlying the results available to ensure full reproducibility?

Yes

Is the study design appropriate and is the work technically sound?

Yes

Are the conclusions drawn adequately supported by the results?

Partly

Are sufficient details of methods and analysis provided to allow replication by others?

Yes

Reviewer Expertise:

Topology Optimization, Design for Additive Manufacturing, Lattice Structure Design, Multi-objective and Multiphysics Optimization.

I confirm that I have read this submission and believe that I have an appropriate level of expertise to confirm that it is of an acceptable scientific standard.

# Reviewer report: Reviewer response for version 1

**DOI:** 10.5256/f1000research.137463.r178994

**Published:** 2023-07-28

**Authors:** Craig M. Hamel

**Affiliations:** 1 Sandia National Laboratories, Albuquerque, Mexico

Review:

 This article describes an approach combining additive manufacturing, FEM, and topology optimization to create rider specific components for motorcycles. While an interesting application and fusion of these technologies, this reviewer found the article to be lacking in several regards that with some modifications can be published. The reviewer’s recommendations are the following

1. The authors should closely check the paper for proper spelling and grammar. There were many locations in the article where a comma could greatly improve readability.
2. Figures 6 and 9 should have their labels or the figure caption modified to point out that the field being plotted is the displacement magnitude.
3. In Figure 10 the authors need to mention which principal strain component they are plotting. Is it the maximum, middle, or minimum principal strain component?
4. A schematic should be provided which shows the mechanism in function from the rider’s hand to the cam. This would allow those who are not experts in the automotive industry to better follow the authors’ work.
5. It would also aid readers to understand what the design inputs from a rider specific perspective are. E.g., how do we need to modify design constraints for a rider with a weaker hand due to injury?
6. Details on the element formulation used in FEM simulations should be provided. The authors mentioned tetrahedral elements, but which tetrahedral element? Additionally, the authors should at least mention mesh convergence and show due diligence on why they choice the specific element size for the specific quantities of interest.
7. The authors do not appear to have considered additional lattice topologies. This addition to the paper would be greatly improve its appeal.

Is the work clearly and accurately presented and does it cite the current literature?

Partly

If applicable, is the statistical analysis and its interpretation appropriate?

Not applicable

Are all the source data underlying the results available to ensure full reproducibility?

Yes

Is the study design appropriate and is the work technically sound?

Yes

Are the conclusions drawn adequately supported by the results?

Yes

Are sufficient details of methods and analysis provided to allow replication by others?

Partly

Reviewer Expertise:

Additive manufacturing, Constitutive modeling

I confirm that I have read this submission and believe that I have an appropriate level of expertise to confirm that it is of an acceptable scientific standard, however I have significant reservations, as outlined above.

# Response

**Published:** 2023-11-15

**Authors:** Marco Freddi

**Affiliations:** University of Bologna, Italy

Dear Reviewer,

 Firstly, thank you for your guidance. We have revised the spelling and grammar of the manuscript.

 Additional specifications have been incorporated into Figures 6 and 9 to facilitate a clearer understanding of the visuals. The same applies to point 3.

 In point 4, you will find a modified image in the manuscript, outlining the assembly of the cam on the handlebar and demonstrating its usage.

 Concerning point 5, we have provided enhanced details at various junctures in the manuscript (highlighted for your convenience).

 Further exploration of lattice topologies will be the subject of forthcoming works that we intend to publish on F1000 Research. Our focus here was to dedicate the manuscript to the methodology used in designing and manufacturing the component.

 Moreover, additional details on the Finite Element Method (FEM) have been added.

 Thank you for your time and consideration.

 Best regards.

# Reviewer report: Reviewer response for version 1

**DOI:** 10.5256/f1000research.137463.r173091

**Published:** 2023-07-28

**Authors:** Osezua Ibhadode

**Affiliations:** 1 Mechanical Engineering, University of Alberta, Edmonton, Alberta, Canada

This work presents a stress-based lattice modeling strategy to redesign a gas cam to become lightweight and retain acceptable structural rigidity. While this design methodology is not entirely new, its application is fresh and should be interesting to the design community. Nonetheless, there are several important points to be addressed by the authors before the work can be accepted. 

1. Highlight the performance of the redesigned cam quantitatively compared to the original cam design.
2. I strongly recommend that you expand the literature mentioned in the introduction. I will suggest exploring at least 5 other related studies. For example, similar work is shown in DOI: 10.5772/intechopen.110371. Include this reference while highlighting the similarities and explain what is different or unique in your work. 
3. It is important to discuss more the original cam, essentially, its function as part of a larger assembly. I also did not find what material is used to make the original cam. Ensure more details about the original cam are included in the revised manuscript.
4. Highlight how you ended up with the design and non-design domains. What informed the decision to keep some regions as non-designable, also mention how you determined the sizes/thicknesses of these domains.
5. nTopology has officially changed its name to nTop. This should be reflected in your revised manuscript. You could also include a software reference. 
6. Was any mesh independence study done? If not, explain how the mesh parameters were chosen to uphold the accuracy of the stress analysis study.
7. In the penultimate row of Table 1, the first column should be "Density after curing".
8. The original gas CAM which is shown in Figure 13 was not numerically analyzed. It is pertinent to compare the stress and displacement results of the original CAM with the redesigned CAM. I strongly recommend this is done and the appropriate comparisons made. Check the work in DOI: 10.5772/intechopen.110371. 
9. What equipment was used to 3D scan the printed cam? Mention the manufacturer and model. Also briefly describe the 3D scanning process. 
10. In the conclusion, quantitative performance comparisons between the original and redesigned cam should be adequately highlighted. 

Is the work clearly and accurately presented and does it cite the current literature?

Yes

If applicable, is the statistical analysis and its interpretation appropriate?

Not applicable

Are all the source data underlying the results available to ensure full reproducibility?

Yes

Is the study design appropriate and is the work technically sound?

Yes

Are the conclusions drawn adequately supported by the results?

Partly

Are sufficient details of methods and analysis provided to allow replication by others?

Yes

Reviewer Expertise:

Topology Optimization, Design for Additive Manufacturing, Lattice Structure Design, Multi-objective and Multiphysics Optimization.

I confirm that I have read this submission and believe that I have an appropriate level of expertise to confirm that it is of an acceptable scientific standard, however I have significant reservations, as outlined above.

## References

[1] : Structural Design Strategies for the Production of Internal Combustion Engine Components by Additive Manufacturing: A Case Study of a Connecting Rod.2022; 10.5772/intechopen.110371 10.5772/intechopen.110371

# Response

**Published:** 2023-11-15

**Authors:** Marco Freddi

**Affiliations:** University of Bologna, Italy

Dear reviewer,

 The performance of the new cam has been prominently highlighted in the final paragraphs of the publication and in the conclusions, particularly concerning the angle of aperture and the reduction in the component's moment of inertia. However, I would like to emphasize that the article's focus lies more on the method rather than solely on the component itself, which serves as a case study example.

 We have included in the literature the work recommended by you, which could serve as a reference point for readers in comparing it with another case study. At the beginning of paragraph 2, you will find a more detailed description of the original component.

 Additionally, we have included a drawing created by us to better illustrate its geometries and functionalities. We have revised the name to 'NTop'.

 Furthermore, in point 4, we have provided improved explanations (all highlighted in the new document). The original cam was not analyzed simply because we do not have its CAD. Our model is new; it draws inspiration from the same cam but is not a redesign of the component itself.

 We have also added information regarding the scanning performed on the component.
